# Multilingual Disparities in Large Language Model–Based Symptom Detection for Global Disease Surveillance: Evaluation Study

**DOI:** 10.2196/103321

**Published:** 2026-10-09

**Authors:** Sa'idah Zahrotul Jannah, Tomohiro Nishiyama, Shaowen Peng, Shoko Wakamiya, Eiji Aramaki

**Affiliations:** 1 Nara Institute of Science and Technology, 8916-5 Takayama-cho, Ikoma, Nara, 630-0192, Japan, 81 743725111; 2 Universitas Airlangga, Surabaya, Jawa Timur, Indonesia

**Keywords:** symptom detection, global disease surveillance, multilingual disparities, large language models, low-resource language, social media

## Abstract

**Background:**

Symptom detection is essential in global disease surveillance to detect potential outbreaks, as symptoms are the first observable signs of infection. To reflect real-time ground truth conditions during pandemics, social media has emerged as a valuable data source. Moreover, effective digital disease surveillance systems must operate across diverse linguistic settings, and large language models (LLMs) have been shown to perform inconsistently across languages, tending to have lower performance in low-resource languages. While multilingual approaches have been explored in various health-related natural language processing tasks, a critical gap remains in understanding whether LLM-based symptom detection can perform consistently across languages for global disease surveillance. Southeast Asia demonstrates this challenge, combining diverse languages and the potential for emerging infectious disease outbreaks, making it a case for evaluating how multilingual performance disparities manifest in symptom detection.

**Objective:**

This study aims to evaluate multilingual disparities in symptom detection using a LLM, as well as the associated error mechanisms across languages and symptom types, to better understand their implications for global disease surveillance.

**Methods:**

This study uses the MedWeb dataset, a multilingual pseudo–social media text dataset with multiple symptom labels. The data consist of 12 languages, covering diverse regions and language resource classifications. The symptoms included in this study are fever, headache, runny nose, cough, diarrhea, hay fever, influenza, and cold. We used GPT-5 as the symptom detection system, representing a strong model for health-related tasks. The results are evaluated using the macro precision, recall, and *F*_1_-score. An error analysis was conducted to identify the error mechanisms underlying incorrect symptom predictions across languages and to calculate the impact of each error category.

**Results:**

Our findings show that performance varied across languages, language resource groups, and symptom types. Southeast Asian (SEA) languages generally achieved lower scores than non-SEA languages, with Japanese obtaining the highest *F*_1_-score (0.812) and Lao the lowest (0.716). High-resource languages achieved the most consistent performance, while low-resource languages obtained the lowest overall scores. At the symptom level, diarrhea, headache, cough, and fever showed stable detection across languages, while hay fever, runny nose, influenza, and cold exhibited greater variability. Hay fever showed the widest variability, forming 2 distinct performance clusters aligned with language resource and region classifications. The error analysis revealed 4 misclassification patterns: explicitly mentioned symptoms, cross-lingual variation, symptom overgeneralization, and context misinterpretation. Cross-lingual variation was the most frequent error category, while errors related to explicitly mentioned symptoms showed the largest potential impact on model performance.

**Conclusions:**

Due to performance disparities, achieving more reliable and equitable LLM-based symptom detection from social media text for global disease surveillance would benefit from broader representation in training data for low-resource languages, improved cultural and linguistic sensitivity, and stronger contextual understanding of symptom-related expressions.

## Introduction

Global disease surveillance plays an important role in public health by detecting potential disease outbreaks. Outbreaks can be identified through symptoms, which are often the first observable signs in the human body after infection [1]. Information shared by social media users during an outbreak can reflect ground truth conditions [2]. This makes social media text a potential data source for detecting symptoms in public health surveillance [3-5].

Social media data used for digital disease surveillance operate across diverse linguistic settings, requiring reliable systems capable of processing multilingual data. This is particularly important for symptom detection, as such systems require not only linguistic understanding but also the ability to interpret symptom expressions across languages. However, the availability of language resources varies across languages. In natural language processing (NLP), languages can be classified along a spectrum ranging from high resource to low resource, depending on factors such as the availability of labeled and unlabeled data [6]. Previous studies have reported that large language models (LLMs) tend to perform better on high-resource languages [7,8].

While prior studies have explored multilingual approaches in health-related NLP tasks, several critical gaps remain. Previous work has evaluated language models’ performance across multiple languages in medical question-answering from clinical text [9], classification of epidemiological characteristics of infectious disease outbreaks [10], symptom entity recognition from clinical text [11], and adverse drug reaction detection [12]. A previous study evaluated LLM-based symptom detection across 7 languages, showing that, on average, European languages outperformed Asian languages and that influenza was significantly overpredicted across all languages [13]. However, these studies did not evaluate how detection performance varies across a broader range of language resource levels, particularly for low-resource languages in the context of disease surveillance. This leaves a gap in understanding whether LLM-based symptom detection systems can perform consistently across diverse language resource levels for global disease surveillance. This gap is particularly critical in regions characterized by linguistic diversity and limited language resources, where the population is also at risk of becoming the epicenter of disease outbreaks.

Southeast Asia presents this challenge, as it comprises diverse midresource to low-resource languages and exhibits relatively lower model performance in these languages [6,14,15]. Moreover, the region has frequently been identified as a source of emerging infectious diseases, highlighting the need for targeted intervention strategies to mitigate outbreak risks [16-19]. This makes Southeast Asia a particularly relevant region for examining symptom detection across diverse regional languages and comparing model performance with higher-resource languages.

Therefore, to address this gap, we specifically evaluate an LLM on a multilingual social media dataset covering 12 languages with diverse resource classifications for multilabel symptom detection. Figure 1 provides an overview of this study: the input is a multilingual, social media–based dataset covering diverse languages and regions, which is then processed by an LLM. The output consists of symptoms detected by the system. We evaluated the results from both language and symptom perspectives, then analyzed the misclassification patterns. Overall, this study design addresses the research objective of evaluating multilingual disparities in symptom detection using an LLM across languages and symptom types to better understand their implications for global disease surveillance. It contributes to identifying multilingual performance disparities and associated error mechanisms in symptom detection, offering insights into the challenge of improving reliability in global disease surveillance systems across diverse languages.

**Figure 1.**
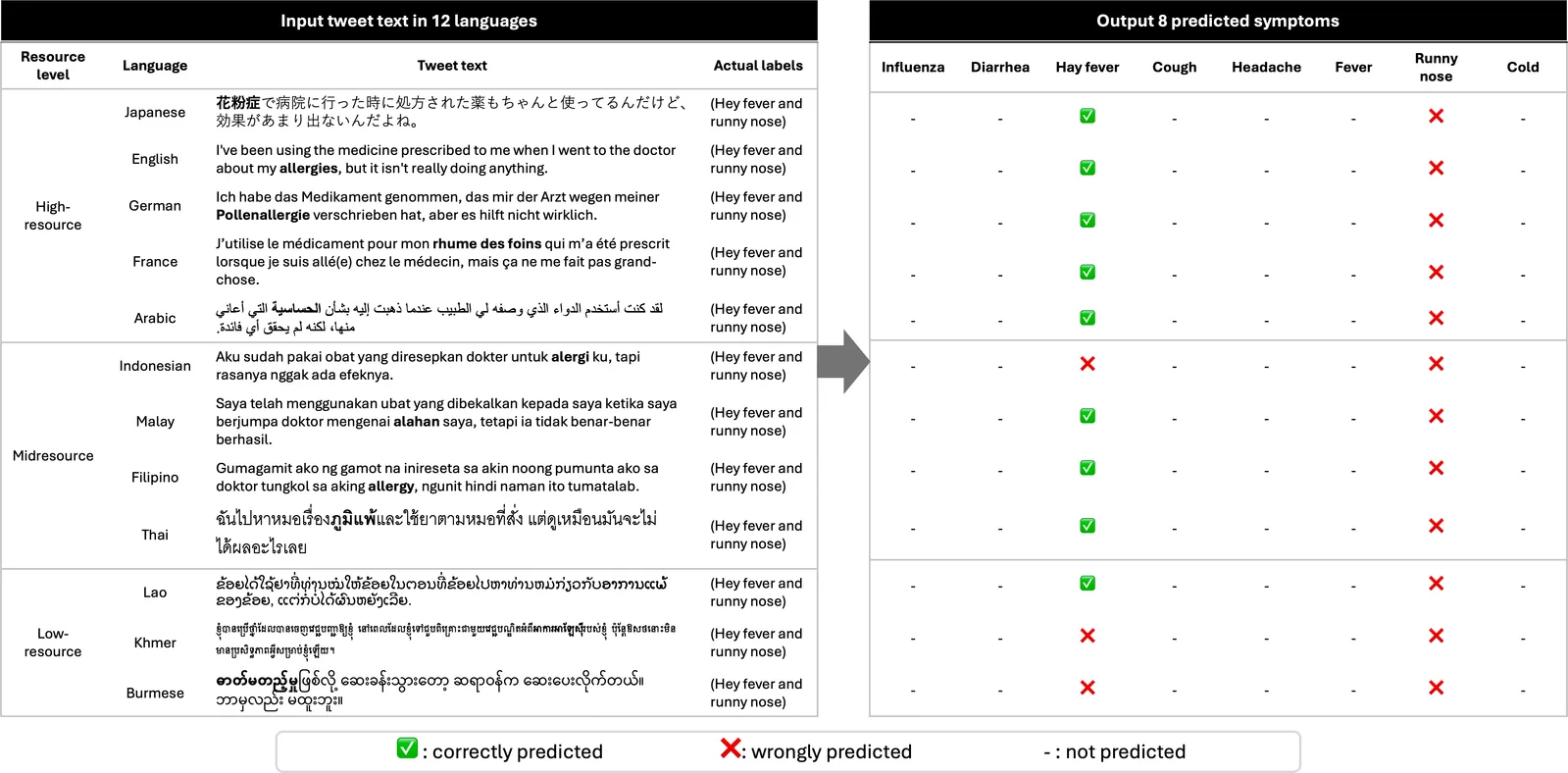
Overview of the study. We evaluate the capability of large language models to predict multilabel symptoms from social media posts across 12 languages, ranging from high-resource to low-resource languages and covering multiple regions. The left panel shows the same social media post across the languages, grouped by language resource level, with the gold symptom labels shown for each text. Bold-highlighted words represent symptom-related words. The right panel shows the model predictions for each symptom and language.

## Methods

### Dataset

This study uses MedWeb, a multilingual pseudo–social media text dataset with multiple symptom labels. The dataset was originally created in Japanese and then human-translated into other languages. The translation-based dataset provides a controlled setting for consistent cross-linguistic comparison of symptom expression. It covers 12 languages, predominantly languages from Southeast Asia (Indonesian, Filipino, Malay, Thai, Khmer, Burmese, and Lao), together with Japanese, English, German, French, and Arabic. Each language contains 640 texts, which are labeled as positive or negative for each symptom or disease (hereafter referred to simply as symptom), allowing multiple positive labels per text [20].

In this study, the languages were categorized by region and resource classification, as shown in Table 1 [6]. The language resource classification was included because LLM performance in NLP tasks is partly influenced by the availability of training data. Therefore, we also investigated whether performance on this symptom detection task differs across languages with varying resource levels.

The study focuses on 8 common symptoms, which are considered indicators associated with other diseases: fever, headache, runny nose, cough, diarrhea, hay fever, influenza, and cold. The label distribution in this dataset is imbalanced between positive and negative labels, with the proportion of positive labels varying across symptoms, as presented in Table 2.

**Table 1.** Language resource classification.

Resource classification	Languages
High	English, German, French, Japanese, and Arabic
Mid	Indonesian, Filipino, Malay, and Thai
Low	Lao, Khmer, and Burmese

**Table 2.** Positive label distribution.

Symptoms	Number of texts with positive labels (N=640)^a^, n (%)
Influenza	24 (3.75)
Diarrhea	64 (10)
Hay fever	46 (7.19)
Cough	80 (12.5)
Headache	77 (12.03)
Fever	93 (14.53)
Runny nose	123 (19.22)
Cold	90 (14.06)

a Percentages are calculated using the total number of texts (N=640) as the denominator for each symptom. As the dataset is multilabel, a text may have zero, one, or multiple positive symptom labels; therefore, label counts and percentages across symptoms are not expected to sum to N or 100%.

### Experimental Settings

We used the GPT-5 (OpenAI) model as a tool for this study. A previous work showed that models from the OpenAI family consistently outperformed others across both large-parameter and small-parameter settings [13]. In addition, GPT-5 is the strongest OpenAI model for health-related questions, achieving higher scores on HealthBench than previous models evaluated in that benchmark [21]. For the prompting strategy, we implemented a rule-based, zero-shot setting in English to reflect a general and realistic deployment scenario. The rules incorporated into the prompt were aligned with the annotation guidelines used during dataset labeling, as shown in Textbox 1.

Textbox 1. **The prompt used in this study.**

**Instruction:**

Determine if the sender of this Twitter message is exhibiting symptoms for each of the following: influenza, diarrhea, hay fever, cough, headache, fever, runny nose, and cold. For each symptom, only answer either 0 or 1 for negative (no symptoms) or positive (has symptoms) respectively. Determination of symptoms is carried out based on the following rules:

- Cases where the symptom is expressed directly, including mild symptoms, are considered positive.
- A symptom can be labeled positive with indirect expressions of a symptom.
- If a symptom is mentioned but then also dismissed or denied, this information is regarded as positive.
- It is considered positive if someone or the user is still affected with such mild symptoms during recovery. However, if the symptoms are completely gone, it is considered negative.
- A symptom is positive even if the user expresses uncertainty regarding its cause.
- Since it is generally presumed that many patients may overlook symptoms or diseases due to insufficient medical knowledge, even suspicion of symptoms and diseases are recognized and labeled positive.
- Symptoms that disappeared completely are recognized and labeled negative. Note that we regarded and labeled positive when a user took medicine that could cause temporary recovery from a symptom.
- For cases that express expectation or process, indicated with words such as “if,” “going,” “if it is,” etc, these should be labeled as negative.
- If the disease is mentioned merely as a topic rather than someone having it, these tweets should be labeled as negative. These include news, general theories, and advertisements.
- If the disease is mentioned in the context of a joke, these should be labeled as negative.
- The symptoms are only for humans.
- Symptoms are within 24 hours, including today.
- The label for symptoms that occurred yesterday is dependent on the disease or symptom.
- Past symptoms, including symptoms 2 or more days ago, are considered negative.
- Recent occurrences and recurring symptoms that still persist are considered positive.
- We regard symptoms in the vicinity and label them as positive regardless of living together or not (ie, family members). We also label symptoms as positive when they are observed from hearsay.
- As for symptoms of people belonging to a specified group in the vicinity (school, club, etc), we labeled them positive.
- Other cases, like symptoms belonging to blog friends, should be labeled as negative since it is difficult to determine their location.

**Post:**

A post in one of the twelve studied languages.

**Output:**

Return the result strictly as a JSON object with the symptoms as keys and the values as either 0 or 1.

We also conducted an ablation study to support the selection of the LLM and the prompting strategy used in the main experiments. For model selection, we evaluated 1 proprietary LLM (GPT-5) and 3 groups of open-weight LLMs: (1) general-purpose models, including Gemma 3 4B (Google DeepMind), Qwen3-VL 8B (Qwen team, Alibaba Cloud), and Llama 3.1 8B (Meta); (2) models fine-tuned for Southeast Asian (SEA) languages, including Gemma-SEA-LION-v4-4B-VL (AI Singapore), Qwen-SEA-LION-v4-8B-VL (AI Singapore), and Llama-SEA-LION-v3-8B (AI Singapore); and (3) models fine-tuned for the medical context, including MedGemma 4B, Bio-Medical-Llama-3-8B, and HuatuoGPT-o1-7B. These models were selected to examine multilingual performance patterns across different model architectures and families and to identify the model with the most suitable performance for further analysis in this study.

In addition to open-weight LLMs, we included supervised models trained on the study dataset and a multilingual zero-shot architecture that was not fine-tuned on the dataset as baselines. These models represented both general-purpose multilingual and medical-domain multilingual approaches across different model paradigms. We further conducted a prompting-strategy ablation to examine the effect of alternative prompt designs. The evaluated strategies included five prompts: (1) rule-based zero-shot setting in English, (2) rule-based zero-shot prompting in each language, (3) rule-based few-shot prompting in English, (4) basic zero-shot prompting in English, and (5) basic zero-shot prompting in each language. This analysis enabled us to assess the contribution of prompt language and structure to model performance.

We further conducted a prompting-strategy ablation to examine the effect of alternative prompt designs. The evaluated strategies included five prompts: (1) rule-based zero-shot setting in English, (2) rule-based zero-shot prompting in each language, (3) rule-based few-shot prompting in English, (4) basic zero-shot prompting in English, and (5) basic zero-shot prompting in each language. This analysis enabled us to assess the contribution of prompt language and structure to model performance.

The complete experimental configurations, including the corresponding results for the evaluated models and prompting strategies, are provided in Multimedia Appendix 1.

### Evaluation Methods

In this study, we analyzed the results from 2 perspectives: a language-based and a symptom-based analysis. Both perspectives used macro precision, recall, and *F*_1_-score. In the symptom-based analysis, the macro *F*_1_-score assigns equal importance to each symptom label, regardless of whether the label is frequent or rare, by calculating the *F*_1_-score for each label separately and then averaging them [22]. This metric provides a clearer picture of the model’s consistency and enables analysis of performance variation across symptoms. Meanwhile, recall measures how many true positive symptom labels the model successfully captures on average for each symptom. In the context of surveillance, recall is important because low recall indicates that the system is missing many true symptom signals. Precision, on the other hand, measures the proportion of predicted positive symptom labels that are correct. In a surveillance system, this is useful for identifying which symptoms generate more false positives, potentially leading to false alarms. This study also conducted an error analysis to identify the mechanisms underlying incorrect symptom predictions across languages. An error tweet was defined as a tweet for which (1) at least one language version contained an incorrect prediction and (2) the prediction patterns were not identical across the language versions. Based on the identified error tweets, we first performed a manual qualitative review to identify recurring error patterns and grouped them into several categories. We then defined each error type and prepared representative examples. Using these definitions and examples, an LLM (GPT-5-mini) was asked to classify all identified error tweets into the corresponding error categories and assign a primary error category to each tweet. Approximately 5% of the LLM-generated classifications were subsequently manually reviewed by a human for quality control. The counterfactual impact analysis was then done by correcting the incorrect predictions in each error tweet based on its primary error category and recalculating the *F*_1_-score. The macro *F*_1_-score was recalculated on the complete multilingual dataset (7680 language-specific texts), with *F*_1_-score calculated for each symptom across all languages and then macroaveraged across the 8 symptom labels. The residual impact of each error type (ΔF_1_) was then measured as the difference between the corrected and original *F*_1_-scores (baseline). A smaller ΔF_1_ means that the error category is causing less remaining performance loss under that prompt.

### Ethical Considerations

This study involved no physical or mental interventions and did not include any experiments requiring human participation. In accordance with the Ethical Guidelines for Medical and Biological Research Involving Human Subjects established by the Japanese government, this study did not require institutional review board approval, as no personally identifiable information was used [23]. The dataset used in this study is publicly available as the extended MedWeb corpus. The original study about the dataset also states that it does not contain personally identifiable information and is exempt from institutional review board approval [20]. Therefore, this study raises no ethical concerns with respect to user privacy or informed consent.

## Results

### Study Findings

Before presenting the main findings, we conducted ablation studies to select the model and prompting strategy. As shown in Figure S1 in Multimedia Appendix 1, among the evaluated LLMs, GPT-5 achieved the highest overall performance with relatively low disparities across languages. In Figure S2 in Multimedia Appendix 1, among prompting strategies, rule-based zero-shot prompting in English showed the lowest performance variability across languages, although its average performance was lower than that of some alternative prompting strategies. Based on these findings, we selected GPT-5 with rule-based zero-shot prompting in English for the main analysis to ensure high and consistent performance across languages.

This section presents the evaluation results of LLM-based multilabel symptom detection across 12 languages, with the aim of examining performance disparities among the languages. The results were analyzed from 2 perspectives: language-level performance and symptom-level performance.

The results of the language-based analysis, as presented in Table 3, showed a noticeable performance gap between SEA languages and the other languages. Overall, SEA languages tend to achieve lower scores across macro *F*_1_-score, precision, and recall than non-SEA languages, with Thai being the only exception. Thai achieved the strongest performance among SEA languages, with scores approaching those of other high-resource languages such as Japanese, English, and French. Among SEA languages, Lao had the lowest *F*_1_-score (0.716) and the widest gap between precision (0.820) and recall (0.658). This indicates that although the model’s predictions are relatively precise, it fails to identify many actual positive cases in the Lao dataset.

**Table 3.** Overall performance metrics across languages, sorted by *F*_1_-score from the highest to lowest^a^.

Language	Resource classification	*F*_1_-score	Precision	Recall
Japanese	High	0.812	0.888	0.770
English	High	0.791	0.873	0.753
French	High	0.788	0.869	0.750
Arabic	High	0.784	0.878	0.736
German	High	0.783	0.873	0.737
Thai	Mid	0.780	0.857	0.742
Malay	Mid	0.735	0.814	0.699
Filipino	Mid	0.731	0.802	0.684
Indonesian	Mid	0.731	0.807	0.689
Khmer	Low	0.728	0.828	0.692
Burmese	Low	0.726	0.826	0.682
Lao	Low	0.716	0.820	0.658

a All metrics indicate that Southeast Asian (SEA) languages tend to perform lower than the others.

This finding is consistent with the ablation study in Multimedia Appendix 1. Figure S1 in Multimedia Appendix 1 generally shows lower performance for SEA languages than for non-SEA languages across LLMs, except for the supervised models. Moreover, this performance gap persists even in models specifically fine-tuned for SEA languages.

To further investigate the disparities, we analyze the performance differences across language resource classification groups, as shown in Figure 2. High-resource languages achieved the highest and the most consistent performance, with Japanese achieving the highest score and appearing as an outlier. The midresource group exhibited greater variability, with Thai standing out as a high-performing outlier, as its performance is closest to that of the high-resource group, while the remaining languages within the group show relatively similar performance levels. Low-resource languages showed the lowest performance among all groups, with Lao obtaining the lowest *F*_1_-score among languages. These results indicate performance disparities across the resource language classification.

**Figure 2.**
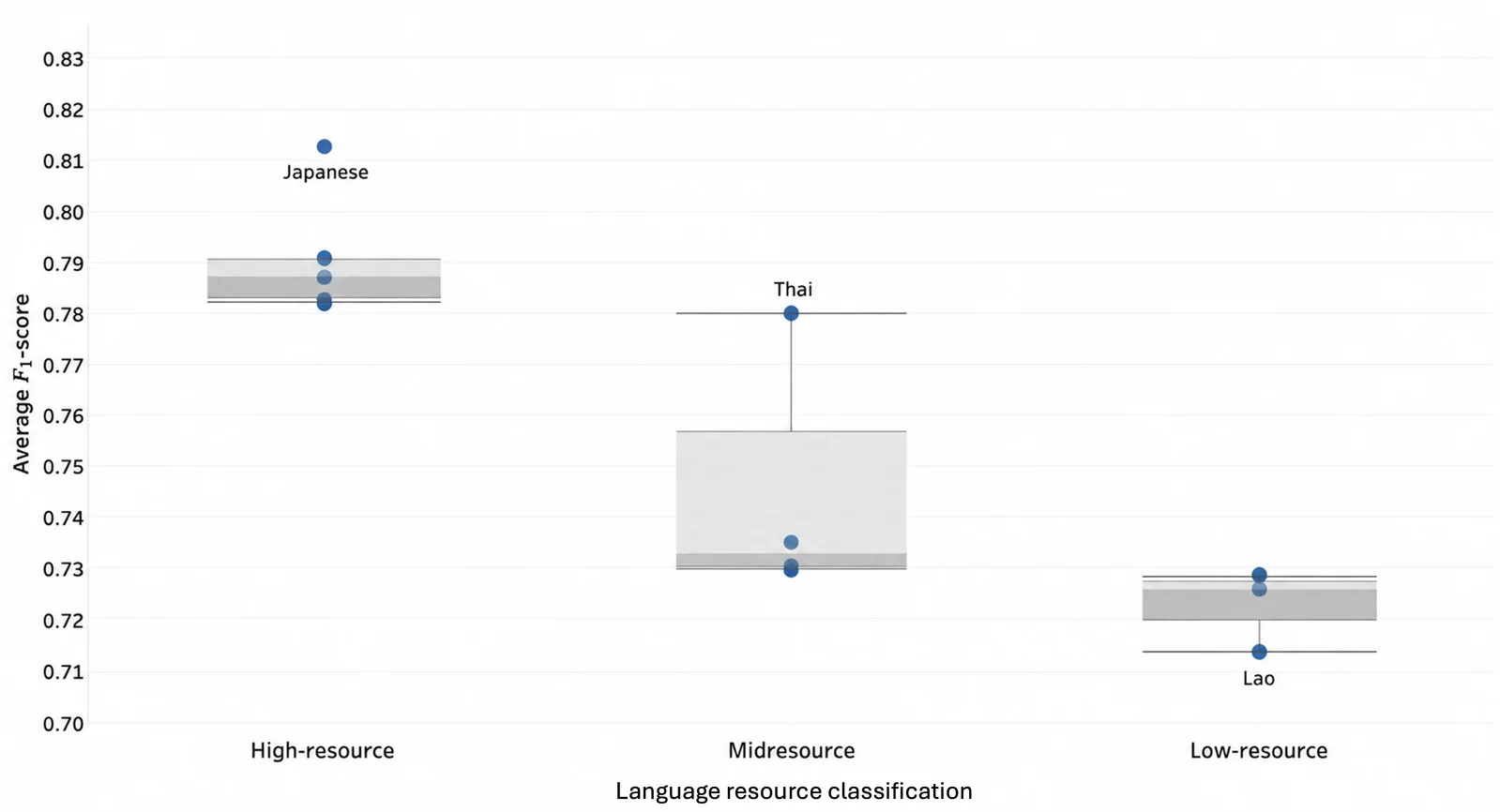
Distribution of average *F*_1_-score across language resource categories (High, mid, and low). Labeled data points represent observations that are potential outliers within each category. High-resource languages achieved the highest performance, while midresource and low-resource languages showed lower performance, with low-resource languages obtaining the lowest overall results.

These results align with findings from our model ablation study in Figure S3 in Multimedia Appendix 1. High-resource languages generally achieve better performance than low-resource languages across the evaluated LLMs, although the ranking of individual languages varies by model category. Japanese achieves the highest performance on GPT-5, while English consistently performs best among high-resource languages across the 3 open-weight LLM groups and the zero-shot pretrained classifier.

Furthermore, we analyzed symptom-level data across languages. Figure 3 shows that detection performance varies substantially across symptoms, with average macro *F*_1_-scores ranging from 0.560 (SD 0.046) for runny nose to 0.896 (SD 0.017) for diarrhea. This suggests that the model’s ability to detect symptoms differs across symptom types. Beyond overall symptom-level differences, we further examine how performances vary across languages within each symptom.

**Figure 3.**
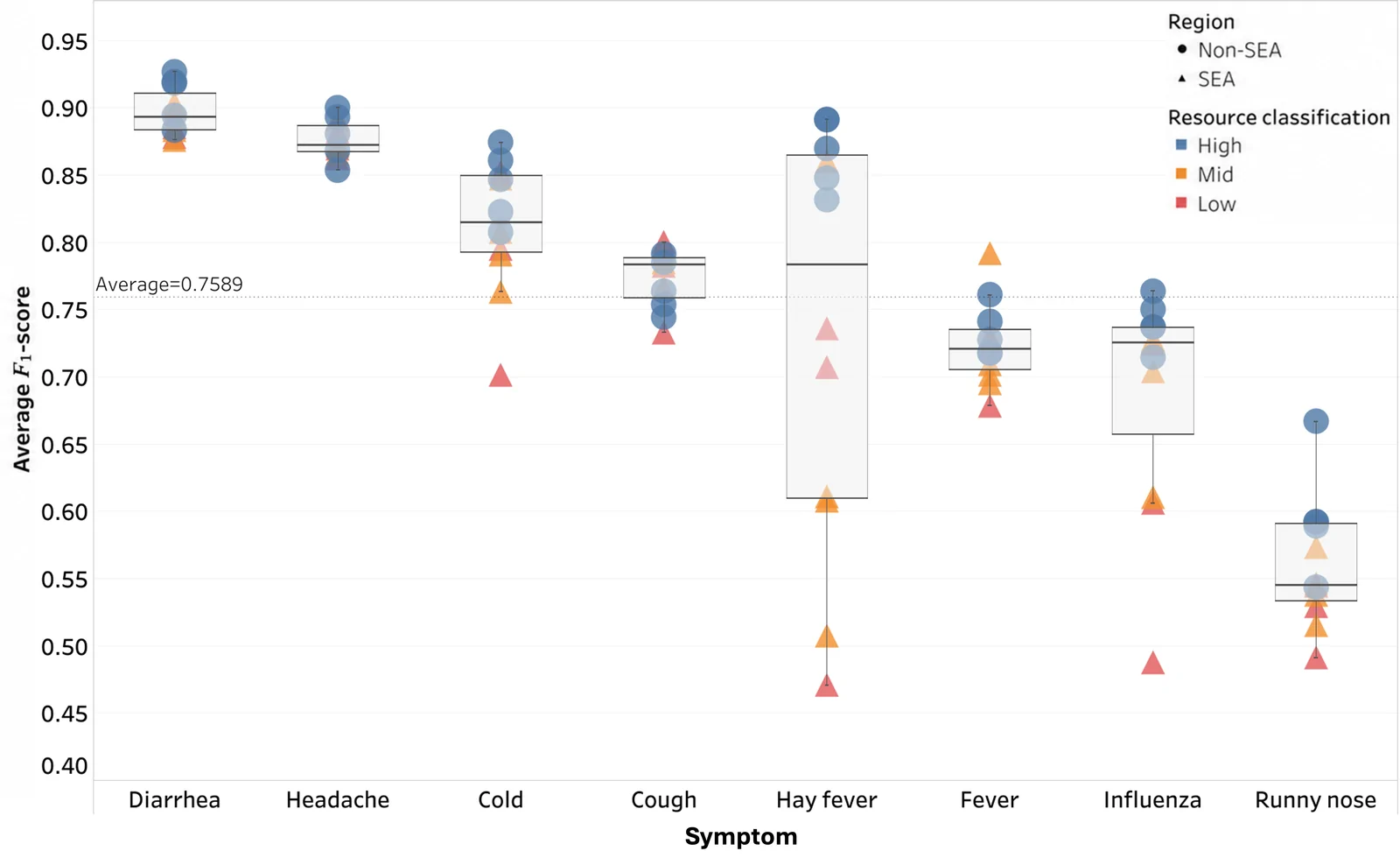
Average macro *F*_1_-score for each symptom across languages, grouped by resource classification and region. High-resource languages tend to achieve higher scores across most symptoms, while greater performance variation is observed in midresource and low-resource languages, particularly for hay fever, where Southeast Asian (SEA) languages show the widest range of scores.

Specifically, there is a notable variation in performance across languages for several symptoms. Diarrhea, headache, cough, and fever showed relatively stable performance across languages. However, cold, runny nose, influenza, and hay fever exhibit large performance variations. Most notably, hay fever detection reveals 2 distinct performance clusters. The first cluster shows high scores, with an average macro *F*_1_-score of 0.865 (SD 0.024). The second cluster exhibits lower and more varied performance, with an average macro *F*_1_-score of 0.607 (SD 0.105). All languages in the low-performance cluster are SEA languages, and these include midresource and low-resource languages. In contrast, the high-performance cluster consists predominantly of high-resource languages, with Thai being the only SEA language in this group. Despite this variation, a consistent pattern emerges that SEA languages classified as midresource to low-resource tend to be placed lower. This indicates a performance gap between languages for some symptoms.

However, an interesting pattern emerged from the experiments with different prompting strategies, as shown in Figure S4 in Multimedia Appendix 1. Few-shot prompting appears to improve the model’s understanding of certain symptoms, particularly hay fever, fever, and runny nose, while no significant differences are observed for the other symptoms. Compared with the basic prompt, however, some symptoms show decreased performance, including diarrhea, hay fever, cough, fever, influenza, and runny nose.

Furthermore, we analyzed precision and recall at the symptom level to understand how the balance between these 2 metrics shapes implications for global disease surveillance systems, particularly for potential outbreak detection. Table 4 showed that several symptoms exhibit an imbalance between their recall and precision scores, with most symptoms having lower recall compared to their precision. Runny nose demonstrated the greatest disparity between the 2, with recall considerably low at 0.427 and precision relatively high at 0.858. Meanwhile, influenza was the only symptom where precision scored lower than recall, indicating that the model tends to overpredict influenza.

**Table 4.** Recall and precision score at the symptom level.

Symptoms	Average of recall (SD)	Average of precision (SD)
Diarrhea	0.857 (0.023)	0.940 (0.017)
Headache	0.903 (0.019)	0.851 (0.014)
Cold	0.742 (0.054)	0.906 (0.066)
Cough	0.645 (0.023)	0.968 (0.017)
Hay fever	0.685 (0.209)	0.826 (0.057)
Fever	0.667 (0.052)	0.797 (0.037)
Influenza	0.806 (0.129)	0.613 (0.059)
Runny nose	0.427 (0.059)	0.858 (0.149)

### Error Analysis

#### Overview of Error Patterns and Impact

In analyzing symptom performance variability, we observed several recurring error patterns. This qualitative error analysis began by examining prediction patterns in a subsample of the dataset. The identified errors were then grouped into four categories: (1) explicitly mentioned symptoms, (2) cross-lingual variation, (3) symptom overgeneralization, and (4) context misinterpretation. Each error type is illustrated using representative tweets and their corresponding prediction errors, as shown in Table S3 in Multimedia Appendix 2. An LLM was subsequently used to classify the identified error tweets into 1 or more of these categories, and a counterfactual impact analysis was subsequently conducted for each primary error category.

Counterfactual impact analysis was conducted across different prompting strategies, all using rule-based prompts, to examine how prompt design affects error patterns and their impact on performance. As shown in Table 5, few-shot prompting in English achieved the greatest improvement. Compared with zero-shot prompting in English, the baseline macro *F*_1_-score increased by 0.0486, from 0.7613 to 0.8099, while the number of error tweets decreased from 296 to 270, a reduction of 26 (8.78%) cases. In contrast, zero-shot prompting in the local language produced only a modest improvement in overall performance.

**Table 5.** Counterfactual impact of primary error categories across rule-based prompting strategies^a^.

Prompt	Error tweets	Baseline *F*_1_-score	Corrected *F*_1_-score (ΔF_1_)			
			Explicitly mentioned symptoms	Cross-lingual variation	Symptom overgeneralization	Context misinterpretation
Rule-zero-English	296	0.7613	0.8547 (+0.0934)	0.8085 (+0.0472)	0.7738 (+0.0125)	0.8023 (+0.0410)
Rule-based few-shot English	270	0.8099	0.8531 (+0.0432)	0.8527 (+0.0428)	0.8206 (+0.0107)	0.8454 (+0.0355)
Rule-zero-local	293	0.7707	0.8515 (+0.0808)	0.8095 (+0.0388)	0.7782 (+0.0075)	0.8035 (+0.0328)

a Values in parentheses indicate the change in macro *F*_1_-score (Δ*F*_1_) after correcting prediction errors in tweets assigned to each primary error category.

#### Explicitly Mentioned Symptoms

Errors in this category occur when a model’s predictions depend on whether a symptom is explicitly expressed in the text. For example, in Case ID 2512, a tweet labeled as influenza and fever is predicted only as influenza in the English dataset, indicating a missed symptom. In contrast, in the Malay version, fever is detected due to the presence of the word *demam*, which corresponds to fever in English. This example shows that the model’s predictions are related to the terms explicitly mentioned in the text.

Based on the error impact analysis, explicitly mentioned symptoms had the greatest impact on model performance across all 3 prompting strategies. Under zero-shot prompting in English, correcting this error category resulted in a +0.0934 increase in macro *F*_1_-score, which was nearly twice the impact of cross-lingual variation or context misinterpretation. With few-shot prompting, the potential impact decreased to +0.0432*;* meanwhile*,* under zero-shot prompting in the local language, it remained relatively high at +0.0808. These findings suggest that providing examples in the prompt may help the model recognize symptom expressions more effectively than simply using the local language for the detection instructions.

#### Cross-Lingual Variation

This error type arises from cross-lingual variation, as differences in how symptoms are expressed across languages can lead to different prediction outcomes. *Demam selesema* in Malay can refer to influenza or a cold, which may lead to confusion between these labels. Another example is shown in case ID 2045, where a tweet labeled as cold is predicted as runny nose in the Filipino and Indonesian datasets. This occurs because *sipon* in Filipino and *pilek* in Indonesian can refer to both runny nose and cold, contributing to misclassification.

Cross-lingual variation was the most frequent error type across all rule-based prompting strategies. However, the impact analysis showed that changing the prompting strategy only modestly reduced its impact. The potential impact of cross-lingual variation was slightly lower with local-language prompting (+0.0388) than with English zero-shot prompting (+0.0472), suggesting that local-language instructions may partially reduce language-specific errors but do not fully address cross-lingual variation.

#### Symptom Overgeneralization

In addition to errors arising from the terms used in the text, the model also shows limitations in understanding context and in disease-related or symptom-related concepts. This is reflected in cases of symptom overgeneralization, where the model predicts additional symptoms commonly associated with the expressed condition but not explicitly stated. For example, in case ID 2119, a tweet labeled as cold and headache is additionally predicted as fever in both the English and Malay datasets.

Based on the impact analysis, this error category had a smaller effect on overall *F*_1_-score performance than the other 3 categories. This is reflected in the consistently smallest difference between the corrected and baseline *F*_1_-scores across all evaluated prompting strategies. Moreover, the relatively similar impact across prompting conditions suggests that modifying the prompt alone may have limited influence on this type of error.

#### Context Misinterpretation

Moreover, context misinterpretation is observed when the model predicts a symptom based solely on its mention without considering the surrounding context. For example, in case ID 2165, the word “flu” appears in the text, but the overall context indicates that the individual does not have influenza. In the English and Arabic datasets, the model correctly predicts the absence of influenza, but in the German and French datasets, it incorrectly predicts the presence of influenza.

The impact analysis showed that contextual errors persisted across all prompting strategies. This suggests that neither providing examples in the prompt nor using the local language was sufficient to fully address context misinterpretation.

## Discussion

### Principal Findings

#### Performance Overview

This study examined the performance gaps in symptom detection tasks using a general-purpose LLM. Based on the 2 perspectives of analysis, language-based and symptom level, our findings showed that performance varies across languages, their resource classifications, and symptom levels.

#### Language-Based Analysis

In this study, language-based disparities were observed between SEA and non-SEA languages. This regional pattern reflects the languages included in each group and their corresponding resource availability rather than indicating that the geographic region itself determines model performance. Most SEA languages in this study are classified as midresource to low-resource, while most non-SEA languages are high-resource. Previous studies have reported similar results regarding performance disparities between English and non-English languages in medical applications of LLMs [24-26], indicating that multilingual performance differences are closely related to how well individual languages are represented and supported in the model.

This interpretation is also consistent with the performance of individual languages. In the GPT-5 experiment, Japanese achieved the highest performance, which may reflect the fact that the Japanese dataset is the original version and therefore preserves the source linguistic context without translation. In contrast, English consistently achieved the highest performance across the other open-weight LLMs. This suggests that the relative advantage of Japanese or English is model-dependent, where Japanese may benefit from being the source language of the dataset and English may benefit from stronger representation in the multilingual training data of some models. Nevertheless, both languages generally outperform the midresource and low-resource languages.

More broadly, previous studies have shown that multilingual LLM performance can be influenced by factors such as pretraining data size and the availability of language resources [27,28]. This is consistent with our findings, which show that high-resource languages generally outperform low-resource languages. Since region and resource level overlap in this study, the lower performance observed for SEA languages is better interpreted as a language-related and resource-related disparity rather than a regional effect. This distinction is essential because SEA is characterized by substantial linguistic diversity and includes many languages with relatively limited NLP resources, and the region is also highly relevant to infectious disease surveillance.

#### Symptom-Based Analysis

We further examine performance differences across symptoms. Our findings indicate that performance varies by symptom, with some symptoms showing relatively consistent performance across languages, while others exhibit substantial variation. Symptoms with low cross-lingual variation, such as diarrhea, headache, cough, and fever, indicate that they are consistently expressed and identified easily across languages. Their lower cross-lingual variability may reflect more consistent symptom expressions or easier model recognition across the evaluated languages.

In contrast, symptoms such as cold, hay fever, influenza, and runny nose exhibit greater performance variability, indicating less stable detection across languages. The model may identify these symptoms well in some languages but not in others. Additionally, compared to low-variation symptoms, these symptoms often overlap with other related symptoms, making them more difficult to detect [29-31]. From a linguistic perspective, this variability also suggests that model performance may depend on the availability of training data and the familiarity with the symptom or disease concept within each language.

Moreover, the error analysis identifies the types of symptom misclassifications. The results indicate that the model relies on lexical cues in the text, while cross-lingual variation in terminology is associated with differences in prediction outcomes. Beyond these errors, the model also predicts additional symptoms associated with the expressed condition even when they are not explicitly mentioned and misclassifies cases in which symptom-related terms are present but negated by the broader context. These findings indicate that the model has difficulty handling implicit meaning, language-specific symptom expressions, and understanding disease concepts.

In terms of frequency, cross-lingual variation was the most common error type. However, the impact analysis showed that the greatest potential performance loss was due to the model’s difficulty in interpreting implicit symptom expressions. Few-shot prompting appeared to reduce both types of errors, particularly the explicitly mentioned symptom errors, while zero-shot prompting in the local language produced only limited improvement. These findings suggest that prompt design alone may not be sufficient to fully address the challenges of multilingual symptom detection.

### Interpreting Hay Fever Variability

Since the primary focus of this study was to analyze disparities in symptom detection performance, we further examined hay fever as a representative symptom, as its detection performance appeared to vary substantially across languages. Hay fever formed 2 distinct clusters of high-performing and low-performing languages, with most SEA languages grouped in the lower-performing cluster. This pattern suggests that some languages support more accurate detection of hay fever, while others may be less capable of capturing hay fever–related terminology or symptom expressions.

This finding emerges alongside regional differences in hay fever–related research and reported cases. Hay fever or allergic rhinitis has been more frequently reported and discussed in regions such as Europe, North America, the Mediterranean, and Japan, while related studies and reported cases remain limited in Southeast Asia [32-36]. As a result, hay fever may be more clearly defined and more frequently referenced in the languages of those regions compared with those used in Southeast Asia. Meanwhile, in LLM-based systems, LLM training data likely reflect the availability of published literature and web content. Therefore, symptoms that are underrepresented in certain language text corpora will be less recognized by the model, causing those symptoms to be more difficult to predict consistently.

This interpretation is also consistent with the ablation study, where providing the model with examples of hay fever cases through few-shot prompting substantially improved its detection performance. This suggests that increasing the model’s familiarity with symptom-related expressions can improve its ability to recognize hay fever across languages. Therefore, the lower performance observed in some languages could be associated with limited exposure to hay fever–related terminology and expressions.

### Implications for Global Disease Surveillance

This study has important implications for the use of LLMs in public health surveillance. Symptom detection is a crucial step in digital surveillance systems, as it enables the identification of health-related signals. However, in global applications involving multiple languages, language-related performance disparities may prevent the model from capturing signals consistently across populations. For example, our findings showed that the model achieved low recall for Lao, indicating that many actual symptom labels in the Lao dataset were missed. As a result, the symptoms from that population could be underdetected. This could lead to certain populations being underrepresented in monitoring and analysis, particularly among populations that are vulnerable to emerging infectious diseases.

In addition to language disparities, performance variation was also observed across symptom types. Large performance differences across symptoms may lead to false alarms or missed signals in surveillance systems. For example, based on our findings, runny nose showed the largest gap between precision and recall, with relatively low recall, suggesting that the system may fail to capture many true runny nose signals. In contrast, influenza was the only symptom for which precision was lower than recall, indicating a greater tendency toward false positive predictions. This may result in influenza-related signals being overdetected in regions without actual outbreaks, potentially leading to unnecessary public concern and inefficient allocation of public health resources.

Furthermore, our findings suggest that the model tends to rely on lexical cues in symptom-related text, while still requiring contextual understanding of diseases and the ability to handle diverse symptom expressions. Therefore, LLM-based symptom-detection systems used in public health surveillance should account for these limitations to ensure consistent and reliable performance. This is particularly important when deploying such systems across diverse linguistic and epidemiological contexts.

### Limitations

This study has several limitations. First, although many symptoms commonly experienced by individuals may serve as signals of other diseases, this study included only 8 symptoms and diseases as representatives. Second, this study uses a translation-based multilingual dataset, which may not fully reflect natural language use in each target language, even though the translations were performed by native speakers. Third, this study uses a limited set of languages and includes Southeast Asia as a representative region that is vulnerable to emerging infectious diseases. However, we acknowledge that other regions may also serve as potential origin points for future outbreaks. Expanding the analysis to a broader range of geographical regions would provide a more comprehensive understanding of global disparities. Fourth, the error analysis is based on qualitative methods in the initial observations, which may not capture all possible sources of model failure. Further research should address these limitations to provide a more comprehensive understanding of potential disparities in symptom detection performance for public health surveillance systems.

### Conclusions

This study demonstrates the presence of multilingual performance disparities in LLM-based symptom detection and offers insights into developing a reliable global disease surveillance system that operates across diverse languages. We identified 3 main points from the study’s findings. First, detection performance tends to vary with language resource level. The observed performance gap between high-resource and low-resource languages, which could be associated with differences in language resource availability, could contribute to underdetection of symptoms in linguistically underrepresented populations, particularly those who are vulnerable to emerging infectious diseases. Second, not all symptoms are equally detectable across languages. While some symptoms are reliably detected across languages, others remain inconsistently identified due to linguistic variability, symptom overlap, and training data familiarity with the symptoms. Third, the error patterns suggest limitations in the model’s ability to detect symptoms from social media text. The current model’s tendency to rely on lexical cues, along with limited contextual and disease-level understanding, represents a challenge for the LLM-based symptom detection systems to identify consistently across diverse languages and symptom expressions. Few-shot prompting may improve the recognition of implicit symptom expressions; however, prompting alone does not fully address broader symptom understanding or contextual interpretation. Therefore, to achieve more reliable and equitable LLM-based symptom detection from social media text for global disease surveillance, it would benefit from broader representation of training data for low-resource languages, improved cultural-linguistic sensitivity, and stronger contextual understanding of symptom-related expressions.

## Supplementary material

10.2196/103321 Multimedia Appendix 1 Ablation studies.

10.2196/103321 Multimedia Appendix 2 Error analysis.

## Abbreviations

LLM: large language model
NLP: natural language processing
SEA: Southeast Asian
