# Supplementary material for: Multilingual Disparities in Large Language Model–Based Symptom Detection for Global Disease Surveillance: Evaluation Study
Source: JMIR AI. 2026 Oct 9;5:e103321. doi: 10.2196/103321 (PMC13652523; doi:10.2196/103321)
Supplement: Supplementary file 1 [file ai-v5-e103321-s001.pdf]

Multimedia Appendix 1: Ablation Studies

Appendix 1.1. Performance of LLM families and supervised baseline models across the languages

We tested ten models, including one proprietary LLM (GPT-5) and three groups of open-weight LLMs: (1) general-purpose models, including Gemma 3 4B, Qwen3-VL 8B, and Llama 3.1 8B; (2) models fine-tuned for Southeast Asian languages, including Gemma-SEA-LION-v4-4B-VL, Qwen-SEA-LION-v4-8B-VL, and Llama-SEA-LION-v3-8B; and (3) models fine-tuned for medical context, including MedGemma 4B, Bio-Medical-Llama-3-8B, and HuatuoGPT-o1-7B. For the fine-tuned LLMs, we selected models that use the corresponding general-purpose LLMs as their base models. For example, Gemma-SEA-LION-v4-4B-VL and MedGemma 4B are built from Gemma 3. This selection allows us to examine the impact of fine-tuning on the target data while controlling for the underlying base model.

For the baseline models, we considered two categories: supervised models and a zero-shot pretrained classifier. The supervised baselines included both general multilingual and medical multilingual models. The general multilingual models consisted of XLM-R, mmBERT, and mT5, while the medical multilingual models included Medical mT5 and KBioXLM. As a zero-shot pretrained classifier, we evaluated GLiNER2 without task-specific fine-tuning on the study dataset. This experimental setup enables us to compare models that learn directly from the labeled dataset through supervised training with a pretrained classifier that performs the task in a zero-shot setting.

The results show that the performance difference between Southeast Asian languages and other languages is generally consistent across the evaluated LLMs, with a similar pattern observed across language resource classifications. In contrast, this trend is not consistently observed among the supervised baseline models. Although some supervised models achieve higher performance than the LLMs, their performance varies substantially across model architectures and languages. Moreover, GLiNER2 achieved lower performance than the instruction-following LLMs, indicating that the evaluated multilingual zero-shot pretrained classifier was less effective for this task. Therefore, the evaluated LLMs show more consistent cross-language performance patterns across model families, while the performance of the supervised baselines is more model- and language-dependent. These findings suggest that the observed multilingual performance disparities are not specific to GPT-5 or a single LLM family.

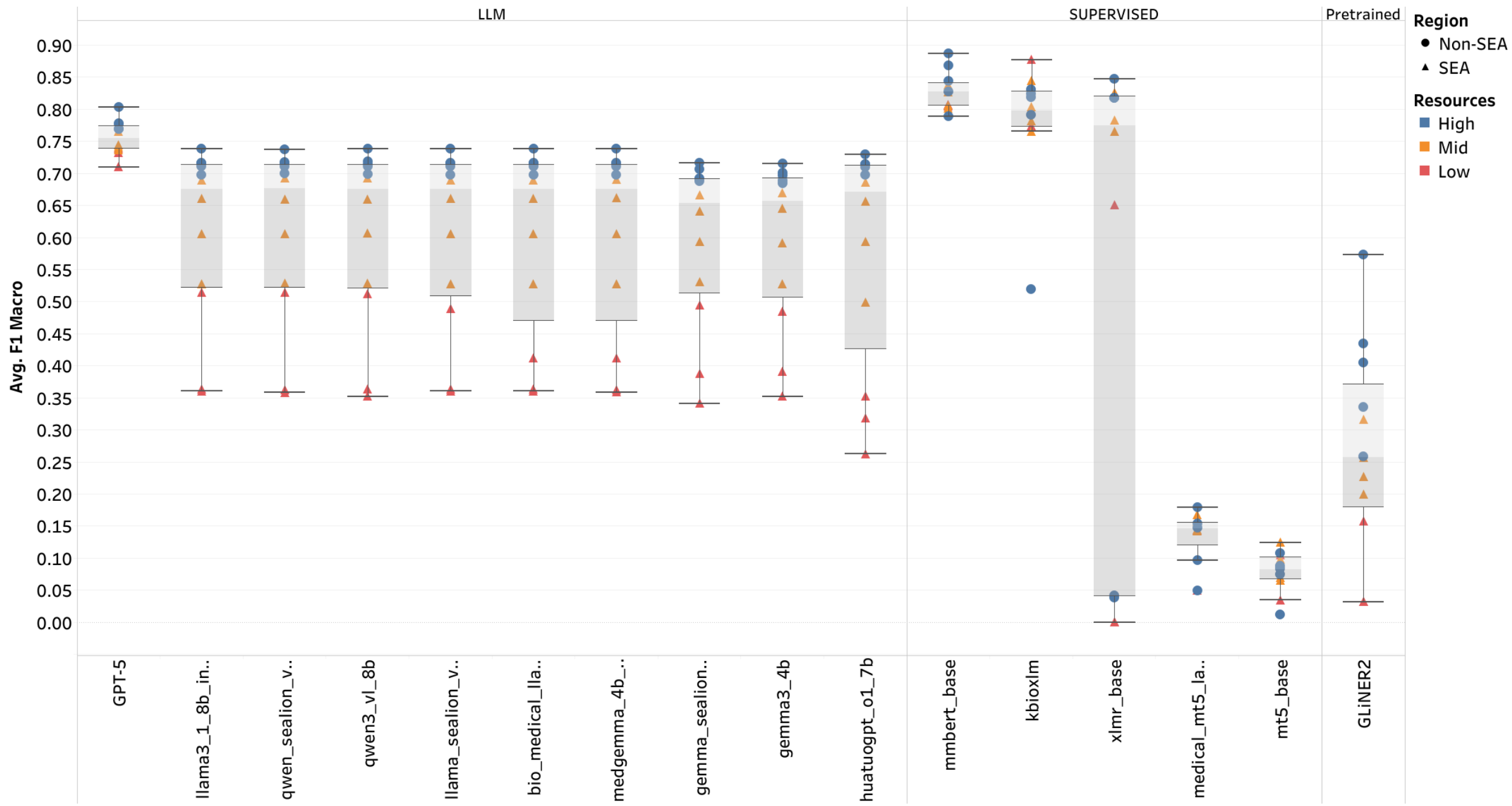

Figure S1. Comparison of the proprietary LLM with open-weight general-purpose, Southeast Asian language–fine-tuned, and medical LLMs across language regions, together with supervised baselines. GPT-5 achieves higher overall performance with lower performance disparities across languages. The open-weight LLM groups show similar cross-lingual performance patterns. Mid- to low-resource Southeast Asian languages generally perform lower than high-resource non-Southeast Asian languages. However, this pattern is not consistently observed for the supervised baseline models.

Appendix 1.2 Sensitivity to prompt design and language localization

In this ablation, we tested five different prompting strategies: rule-based zero-shot prompting in English (rule-zero-english), rule-based zero-shot prompting in each language (rule-zero-local), rule-based few-shot prompting in English (rule-fewshot-english), basic zero-shot prompting in English (basic-zero-english), and basic zero-shot prompting in each language (basic-zero-local). Rule-based prompting refers to using prompts that follow the annotation guidelines. Basic prompting refers to direct instructions or minimal prompting without any predefined rules. Zero-shot means that no examples are provided in the prompt, whereas few-shot means that one example tweet corresponding to each rule is included in the prompt. Detailed prompts are provided in Appendix 1.6

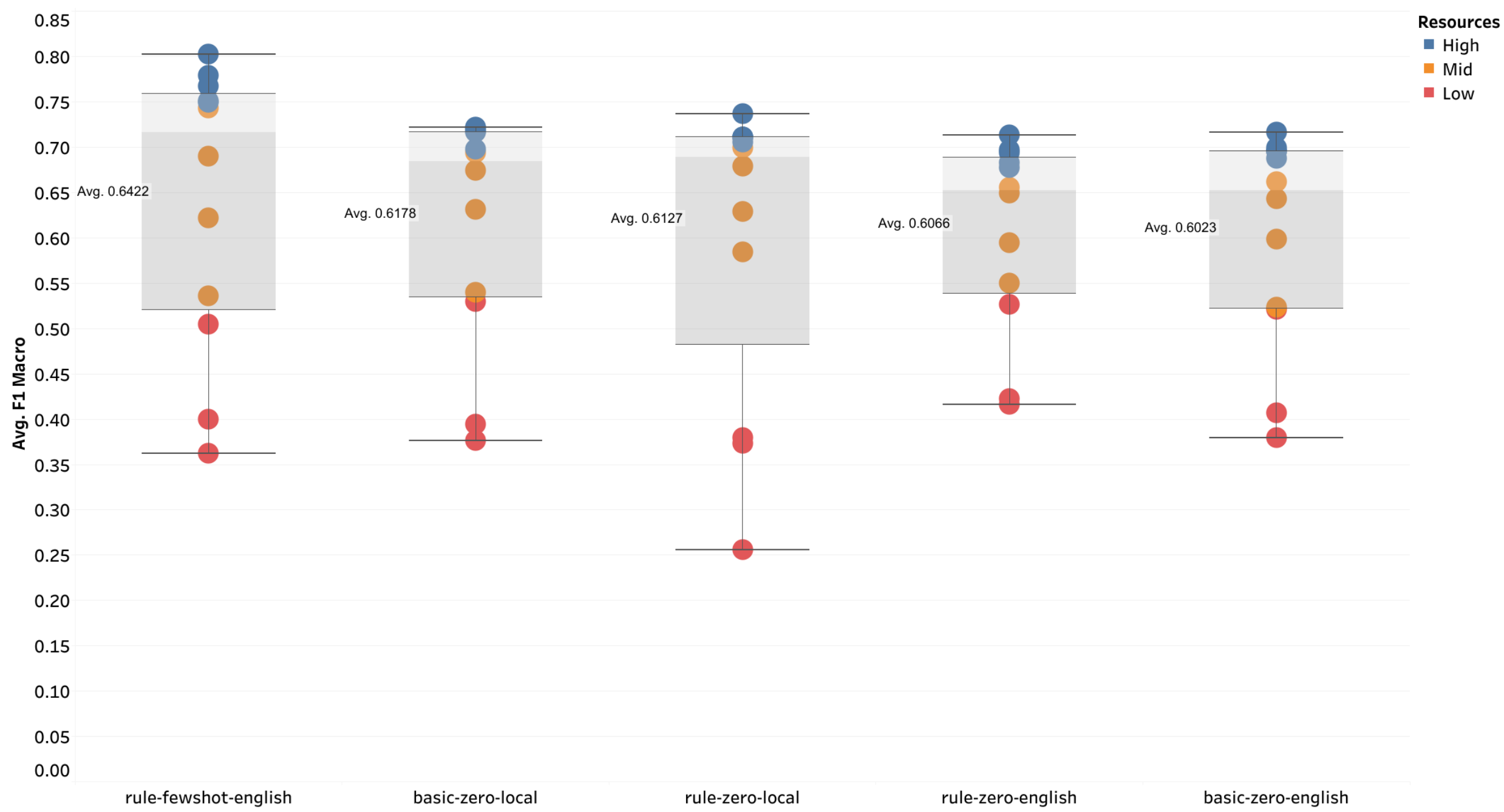

Figure S2. Distribution of prompting strategies across languages and their resource classifications, with the aggregation across the large language models. Rule-based few-shot prompting in English achieves the best performance, while the zero-shot setting shows lower performance but the least variation among the prompting strategies. Prompts using the local language of each target language generally achieve higher performance, except for low-resource languages.

Appendix 1.3 Resource Classification

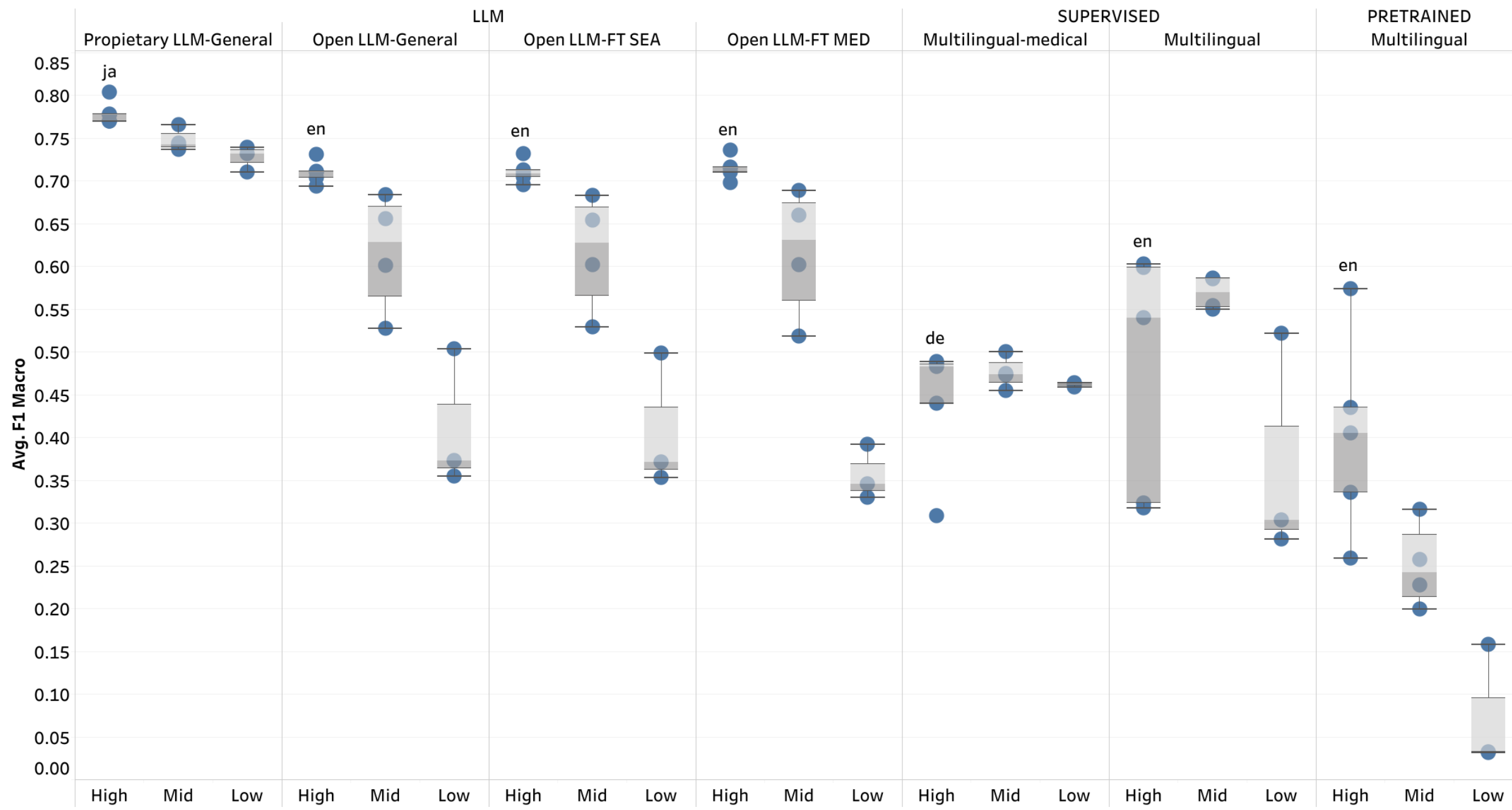

Figure S3. High-resource languages generally achieve higher performance than low-resource languages across the evaluated LLMs and zero-shot pretrained classifier. However, this trend is not observed for the supervised models. Labeled points indicate the best-performing language within each resource classification group for each model category. Japanese achieves the highest performance for the proprietary model, whereas English consistently performs best across the three open-weight model groups and the pretrained classifier model.

Appendix 1.4 Symptom-based analysis

The results show that symptom detection for some symptoms is sensitive to the prompting strategy. Some symptoms achieve better prediction performance with rule-based prompts than with basic prompts, and with few-shot prompting than with zero-shot prompting. In contrast, the performance for Cold remains relatively consistent across prompting settings.

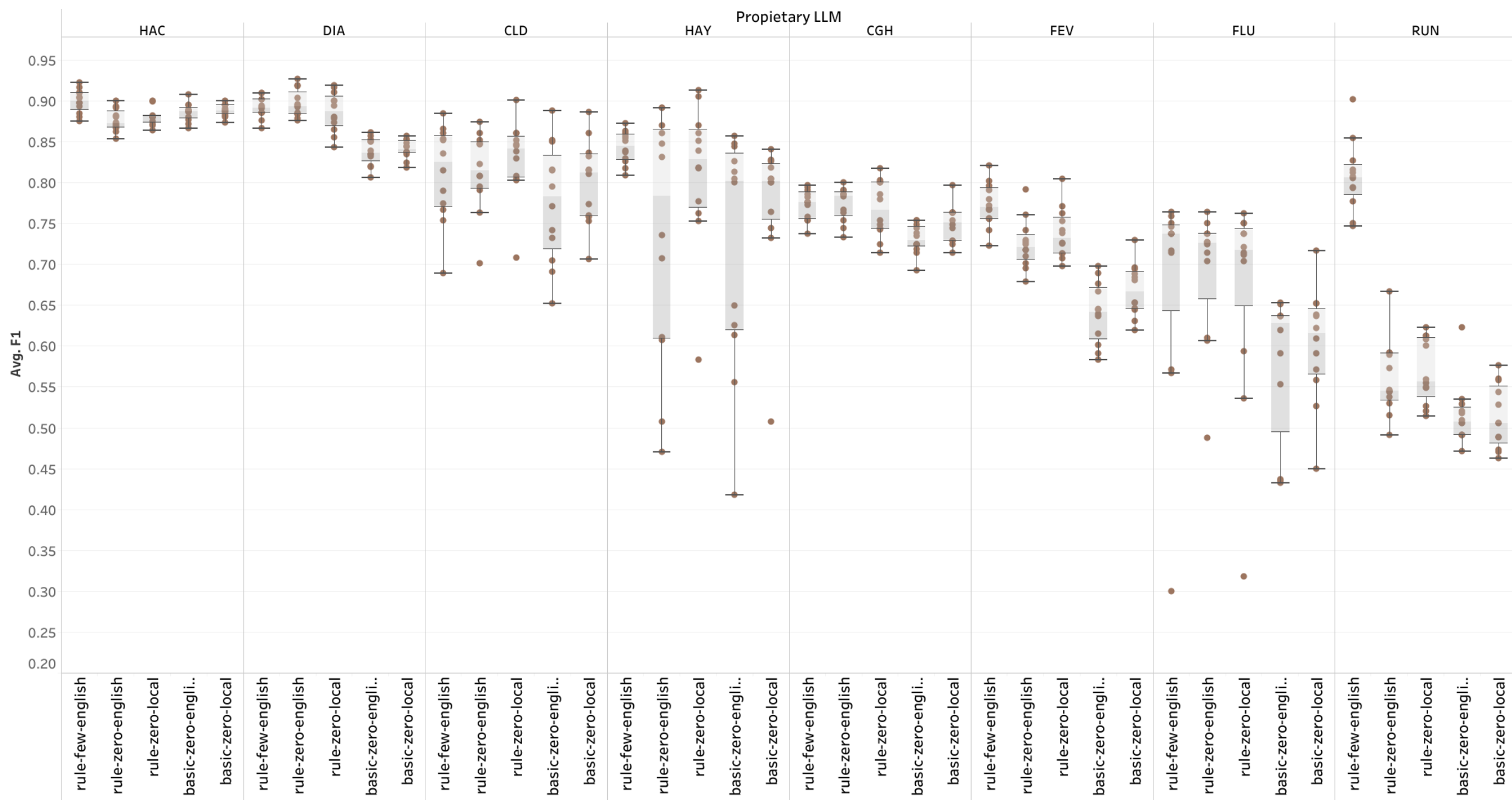

Figure S4. Comparison of prompt settings for GPT-5. Compared with the rule-based zero-shot setting in English, the few-shot setting achieves higher performance, particularly for Hay fever (HAY), Fever (FEV), and Runny nose (RUN), while the differences for the other symptoms are not substantial. For some symptoms, the basic prompt results in lower performance, particularly for Diarrhea (DIA), Hay fever (HAY), Cough (CGH), Fever (FEV), Influenza (FLU), and Runny nose (RUN).

Appendix 1.5 Supervised baseline model settings

Table S1. Supervised baseline model configurations. All models were fine-tuned independently for each language for multilabel classification of eight symptoms. No multilabel stratification in the 80/20 split

Model	XLM-R	mmBERT	KBioXLM	mT5	Medical-mT5
**Architecture**	Encoder-only	Encoder-only	Encoder-only	Encoder-decoder	Encoder-decoder
**Learning Rate**	2.00E-05	2.00E-05	2.00E-05	1.00E-05	3.00E-06
**Epochs**	10	10	10	10	10
**Batch Size**	8	8	8	8	8
**Max Length**	128	128	128	128	128
**Warmup Ratio**	0.1	0.1	0.1	0.1	0.1
**Max Grad Norm**	1	1	1	1	1
**Prediction Threshold**	0.5	0.5	0.5	0.5	0.5
**Train/Test Split**	80/20	80/20	80/20	80/20	80/20
**Random Seed**	42	42	42	42	42

Appendix 1.6 Prompts used for symptom detection

Table S2. Prompts used in the ablation study

Prompt setting	Language	Prompt
rule-zero-english	English	"""Determine if the sender of this Twitter message is exhibiting symptoms for each of the following: {symptoms_str}.<br>For each symptom, only answer either 0 or 1 for negative (no symptoms) or positive (has symptoms) respectively.<br>Determination of symptoms is carried out based on the following rules:<br>Cases where the symptom is expressed directly including mild symptoms are considered positive;<br>A symptom can be labeled positive with indirect expressions of a symptom;<br>If a symptom is mentioned but then also dismissed or denied, this information is regarded as positive;<br>it is considered positive if someone or the user is still affected with such mild symptoms during recovery. However, if the symptoms are completely gone, it is considered negative;<br>A symptom is positive even if the user expresses uncertainty regarding its cause;<br>Since it is generally presumed that many patients may overlook symptoms or diseases due to insufficient medical knowledge, even suspicion of symptoms and diseases are recognized and labeled positive;<br>Symptoms that disappeared completely are recognized and labeled negative. Note that we regarded and labeled positive when a user took medicine that could cause temporary recovery from a symptom;<br>For cases that express expectation or process, indicated with words such as "if," "going," "if it is," etc., these should be labeled as negative;<br>If the disease is mentioned merely as a topic rather than someone having it, these tweets should be labeled as negative. These include news, general theories, and advertisements;<br>If the disease is mentioned in the context of a joke, these should be labeled as negative;<br>the symptoms are only for humans;<br>symptoms are within 24 hours including today;<br>The label for symptoms that occurred yesterday are dependent on the disease or symptom;<br>Past Symptoms Including Two or More Days Ago considered as negative;<br>Recent Occurrence and Recurring Symptom that Still Persists considered as positive;<br>We regarded as a symptom in the vicinity and labeled positive regardless of living together or not (i.e. family members). We also labeld positive when a symptom was observed from hearsay;<br>As for symptoms of people belonging to a specified group in the vicinity (school, club, etc.), we labeled them positive. Other cases, like symptoms belonging to blog friends, should be labeled as negative since it is difficult to determine their location;<br>Tweet: {tweet}<br>IMPORTANT: JSON keys MUST be exactly as listed above: {symptoms_str}. Use these exact names as keys.<br>Return the result strictly as a JSON object with the symptoms as keys and the values as either 0 or 1.<br>Ensure that the output is valid JSON format.<br>Do not tell me what language the tweet is in, just provide the JSON object.<br>Respond with ONLY the JSON object. No explanation, no markdown, no code fences."""

Prompt setting	Language	Prompt
rule-zero-local	Indonesian	"""Tentukan apakah pengirim pesan Twitter ini menunjukkan gejala untuk masing-masing berikut: {symptoms_str}.<br>Untuk setiap gejala, jawab hanya dengan 0 atau 1: 0 untuk negatif (tidak ada gejala) dan 1 untuk positif (ada gejala).<br>Penentuan gejala dilakukan berdasarkan aturan berikut:<br>Kasus di mana gejala diungkapkan secara langsung, termasuk gejala ringan, dianggap positif;<br>Gejala dapat diberi label positif meskipun diungkapkan secara tidak langsung;<br>Jika suatu gejala disebutkan tetapi kemudian disangkal atau ditolak, ini tetap dianggap positif;<br>Dianggap positif jika seseorang atau pengguna masih terpengaruh oleh gejala ringan tersebut selama masa pemulihan. Namun, jika gejala sudah benar-benar hilang, dianggap negatif;<br>Gejala tetap dianggap positif meskipun pengguna menyatakan ketidakpastian mengenai penyebabnya;<br>Karena banyak pasien mungkin mengabaikan gejala atau penyakit karena kurangnya pengetahuan medis, bahkan kecurigaan terhadap gejala dan penyakit diberi label positif;<br>Gejala yang benar-benar hilang diberi label negatif. Catatan: jika pengguna minum obat yang menyebabkan pemulihan sementara dari suatu gejala, ini tetap diberi label positif;<br>Untuk kasus yang menyatakan harapan atau proses, ditandai dengan kata seperti "kalau," "akan," "kalau begitu," dll., ini diberi label negatif;<br>Jika penyakit hanya disebutkan sebagai topik dan bukan sebagai sesuatu yang dialami seseorang, tweet ini diberi label negatif. Ini termasuk berita, teori umum, dan iklan;<br>Jika penyakit disebutkan dalam konteks lelucon, ini diberi label negatif;<br>gejala hanya berlaku untuk manusia;<br>gejala dalam kurun waktu 24 jam termasuk hari ini;<br>Label untuk gejala yang terjadi kemarin bergantung pada jenis penyakit atau gejalanya;<br>Gejala masa lalu termasuk yang terjadi dua hari atau lebih yang lalu dianggap negatif;<br>Kejadian baru-baru ini dan gejala berulang yang masih berlangsung dianggap positif;<br>Gejala yang dialami orang di sekitar dianggap positif terlepas dari tinggal bersama atau tidak (misalnya anggota keluarga). Kami juga memberi label positif jika gejala diketahui dari desas-desus;<br>Untuk gejala yang dialami orang dalam kelompok tertentu di sekitar (sekolah, klub, dll.), kami memberi label positif. Kasus lain, seperti gejala teman blog, diberi label negatif karena sulit menentukan lokasi mereka;<br>Tweet: {tweet}<br>PENTING: kunci (keys) dalam objek JSON HARUS persis seperti yang tercantum di atas: {symptoms_str}. Gunakan nama-nama ini persis sebagai kunci.<br>Kembalikan hasilnya secara ketat sebagai objek JSON dengan gejala sebagai kunci dan nilainya berupa 0 atau 1.<br>Pastikan outputnya berupa format JSON yang valid.<br>Jangan memberi tahu saya bahasa apa yang digunakan dalam tweet ini, cukup berikan objek JSON.<br>Jawab HANYA dengan objek JSON. Tanpa penjelasan, tanpa markdown, tanpa code fences."""
rule-zero-local	Malay	Tentukan sama ada penghantar mesej Twitter ini menunjukkan gejala bagi setiap yang berikut: {symptoms_str}.<br>Bagi setiap gejala, jawab hanya dengan 0 atau 1: 0 untuk negatif (tiada gejala) dan 1

Prompt setting	Language	Prompt
		untuk positif (ada gejala).<br>Penentuan gejala dibuat berdasarkan peraturan berikut:<br>Kes di mana gejala dinyatakan secara langsung, termasuk gejala ringan, dianggap positif;<br>Gejala boleh dilabel positif walaupun dinyatakan secara tidak langsung;<br>Jika gejala disebut tetapi kemudian dinafikan atau disangkal, ini tetap dianggap positif;<br>Dianggap positif jika seseorang atau pengguna masih terjejas oleh gejala ringan itu semasa pemulihan. Walau bagaimanapun, jika gejala telah hilang sepenuhnya, ia dianggap negatif;<br>Gejala tetap dianggap positif walaupun pengguna menyatakan ketidakpastian mengenai puncanya;<br>Oleh kerana ramai pesakit mungkin terlepas pandang gejala atau penyakit disebabkan kurangnya pengetahuan perubatan, walaupun syak wasangka terhadap gejala dan penyakit diberi label positif;<br>Gejala yang telah hilang sepenuhnya diberi label negatif. Perhatikan bahawa jika pengguna mengambil ubat yang menyebabkan pemulihan sementara daripada gejala, ini tetap diberi label positif;<br>Bagi kes yang menyatakan jangkaan atau proses, ditandakan dengan perkataan seperti "jika," "akan," "kalau begitu," dan lain-lain, ini dilabel negatif;<br>Jika penyakit hanya disebut sebagai topik dan bukan sesuatu yang dialami seseorang, tweet ini dilabel negatif. Ini termasuk berita, teori am, dan iklan;<br>Jika penyakit disebut dalam konteks jenaka, ini dilabel negatif;<br>gejala hanya untuk manusia;<br>gejala dalam tempoh 24 jam termasuk hari ini;<br>Label bagi gejala yang berlaku semalam bergantung kepada jenis penyakit atau gejala;<br>Gejala lampau termasuk yang berlaku dua hari atau lebih dahulu dianggap negatif;<br>Kejadian baru-baru ini dan gejala berulang yang masih berterusan dianggap positif;<br>Gejala yang dialami orang di sekeliling dianggap positif tanpa mengira tinggal bersama atau tidak (contohnya ahli keluarga). Kami juga memberi label positif jika gejala diketahui daripada khabar angin;<br>Bagi gejala orang dalam kumpulan tertentu di sekeliling (sekolah, kelab, dll.), kami memberi label positif. Kes lain, seperti gejala rakan blog, dilabel negatif kerana sukar menentukan lokasi mereka;<br>Tweet: {tweet}<br>PENTING: kunci (keys) dalam objek JSON MESTI sama persis seperti yang disenaraikan di atas: {symptoms_str}. Gunakan nama-nama ini dengan tepat sebagai kunci.<br>Kembalikan hasil secara ketat sebagai objek JSON dengan gejala sebagai kunci dan nilai sama ada 0 atau 1.<br>Pastikan output dalam format JSON yang sah.<br>Jangan beritahu saya bahasa apa yang digunakan dalam tweet ini, hanya berikan objek JSON.<br>Jawab HANYA dengan objek JSON. Tiada penjelasan, tiada markdown, tiada code fences."""
rule-zero-local	Filipino	Tukuyin kung ang nagpadala ng mensaheng Twitter na ito ay nagpapakita ng sintomas para sa bawat isa sa mga sumusunod: {symptoms_str}.<br>Para sa bawat sintomas, sumagot lamang ng 0 o 1 para sa negatibo (walang sintomas) o positibo (may sintomas), ayon sa pagkakabanggit.<br>Ang pagtukoy ng sintomas ay batay sa mga sumusunod na patakaran:<br>Ang mga kaso kung saan direktang inihayag ang sintomas, kasama na ang mild na sintomas, ay itinuturing na positibo;

Prompt setting	Language	Prompt
		Maaaring i-label na positibo ang isang sintomas kahit hindi direktang ipinahayag;<br>Kung binanggit ang isang sintomas ngunit pinabulaanan o itinanggi rin, ito ay itinuturing pa ring positibo;<br>Itinuturing itong positibo kung ang user o ang taong tinutukoy ay apektado pa rin ng mild na sintomas habang gumagaling. Gayunpaman, kung ganap nang nawala ang sintomas, ito ay negatibo;<br>Positibo pa rin ang isang sintomas kahit ipinahayag ng user ang kawalang-katiyakan tungkol sa sanhi nito;<br>Dahil karaniwang inaakala na maraming pasyente ang maaaring hindi mapansin ang mga sintomas o sakit dahil sa kakulangan ng kaalamang medikal, kahit hinala lamang sa sintomas o sakit ay kinikilala at ini-label na positibo;<br>Ang mga sintomas na ganap nang nawala ay ini-label na negatibo. Tandaan na itinuturing at ini-label naming positibo kung ang user ay uminom ng gamot na maaaring magdulot ng pansamantalang paggaling mula sa isang sintomas;<br>Para sa mga kasong nagpapahayag ng inaasahan o proseso, tulad ng mga salitang "kung," "papunta na," "kung ito ay," atbp., ito ay dapat i-label na negatibo;<br>Kung ang sakit ay binanggit lamang bilang paksa sa halip na may nakaranas nito, ang mga tweet na ito ay dapat i-label na negatibo. Kasama rito ang balita, pangkalahatang teorya, at mga patalastas;<br>Kung ang sakit ay binanggit sa konteksto ng biro, ito ay dapat i-label na negatibo;<br>ang mga sintomas ay para lamang sa tao;<br>ang mga sintomas ay nasa loob ng 24 oras kasama ang ngayong araw;<br>Ang label para sa mga sintomas na naganap kahapon ay nakadepende sa uri ng sakit o sintomas;<br>Ang mga nakaraang sintomas kasama ang mga naganap dalawang araw o higit pa ang nakalipas ay itinuturing na negatibo;<br>Ang kamakailang pangyayari at umuulit na sintomas na patuloy pa rin ay itinuturing na positibo;<br>Itinuturing naming positibo ang sintomas ng taong nasa paligid, kahit sila ba ay magkasama sa tirahan o hindi (hal. mga miyembro ng pamilya). Ini-label din naming positibo kung ang sintomas ay nabatid lamang sa balita-balita;<br>Para sa mga sintomas ng mga taong kabilang sa isang tiyak na grupo sa paligid (paaralan, klub, atbp.), ini-label namin itong positibo. Ang ibang kaso, tulad ng sintomas ng mga kaibigan sa blog, ay dapat i-label na negatibo dahil mahirap matukoy ang kanilang lokasyon;<br>Tweet: {tweet}<br>MAHALAGA: ang mga JSON key ay DAPAT eksaktong katulad ng nakalista sa itaas: {symptoms_str}. Gamitin ang eksaktong mga pangalang ito bilang keys.<br>Ibalik ang resulta nang mahigpit bilang JSON object kung saan ang mga sintomas ang keys at ang values ay 0 o 1.<br>Tiyaking valid JSON format ang output.<br>Huwag sabihin sa akin kung anong wika ang ginamit sa tweet, JSON object lamang ang ibigay.<br>Sumagot LAMANG ng JSON object. Walang paliwanag, walang markdown, walang code fences.
rule-zero-local	Japanese	以下のそれぞれの症状について、この Twitter メッセージの発信者が症状を示しているかどうかを判定してください: {symptoms_str}。<br>各症状について、陰性（症状なし）は 0、陽性（症状あり）は 1 のみで答えてください。<br>症状の判定は以下のルールに基づいて行います:<br>軽度の症状を含め、症状が直接表現されている場合は陽性とみなす;<br>間接的な表現であっても症状は陽性とラベル付けできる;

Prompt setting	Language	Prompt
		症状が言及された後に否定・否認された場合も、陽性とみなす;<br>回復期にまだ軽い症状の影響を受けている場合は陽性とみなす。ただし、症状が完全になくなっている場合は陰性とみなす;<br>原因について不確かさを表現していても、症状は陽性とみなす;<br>多くの患者は医学知識の不足から症状や病気を見逃す可能性があると一般に考えられるため、症状や病気への疑いだけでも陽性と認識・ラベル付けする;<br>完全になくなった症状は陰性とラベル付けする。なお、薬の服用によって一時的に症状が回復した場合は陽性とラベル付けする;<br>「もし」「これから」「〜だったら」などの言葉で示される期待や予定を表す場合は、陰性とラベル付けする;<br>病気が誰かが罹患しているものとしてではなく、単に話題として言及されている場合（ニュース、一般論、広告など）は陰性とラベル付けする;<br>病気が冗談の文脈で言及されている場合は陰性とラベル付けする;<br>症状は人間に関するもののみを対象とする;<br>症状は本日を含む 24 時間以内のものとする;<br>昨日発生した症状のラベルは、病気や症状の種類によって異なる;<br>2 日以上前の過去の症状は陰性とみなす;<br>最近発生し、まだ持続している再発性の症状は陽性とみなす;<br>同居の有無にかかわらず、身近な人（家族など）の症状も陽性とラベル付けする。また、伝聞で症状を知った場合も陽性とラベル付けする;<br>身近な特定のグループ（学校、クラブなど）に属する人々の症状は陽性とラベル付けする。ブログ友達の症状など、所在を特定しにくい他のケースは陰性とラベル付けする;<br>Tweet: {tweet}<br>重要: JSON のキーは必ず上記の通りにしてください: {symptoms_str}。これらの名称を正確にキーとして使用してください。<br>症状をキーとし、値を 0 または 1 とした JSON オブジェクトとして、結果を厳密に返してください。<br>出力が有効な JSON 形式であることを確認してください。<br>ツイートが何語であるかは伝えず、JSON オブジェクトのみを提供してください。<br>JSON オブジェクトのみで回答してください。説明、マークダウン、コードフェンスは不要です。"""
rule-zero-local	Thailand	"""พิจารณาว่าผู้ส่งข้อความ Twitter นี้แสดงอาการของแต่ละอาการต่อไปนี้หรือไม่: {symptoms_str}<br>สำหรับแต่ละอาการ ให้ตอบเพียง 0 หรือ 1 เท่านั้น โดย 0 หมายถึงลบ (ไม่มีอาการ) และ 1 หมายถึงบวก (มีอาการ)<br>การพิจารณาอาการเป็นไปตามกฎต่อไปนี้:<br>กรณีที่แสดงอาการโดยตรง รวมถึงอาการเล็กน้อย ถือว่าเป็นบวก;<br>อาการสามารถถูกระบุว่าเป็นบวกได้แม้จะแสดงออกทางอ้อม;<br>หากมีการกล่าวถึงอาการแต่ภายหลังปฏิเสธหรือไม่ยอมรับ ให้ถือว่าเป็นบวก;<br>ถือว่าเป็นบวกหากบุคคลหรือผู้ใช้ยังคงได้รับผลกระทบจากอาการเล็กน้อยในช่วงพักฟื้น อย่างไรก็ตาม<br>หากอาการหายไปโดยสมบูรณ์แล้ว ให้ถือว่าเป็นลบ;<br>อาการยังคงเป็นบวกแม้ผู้ใช้จะแสดงความไม่แน่ใจเกี่ยวกับสาเหตุ;<br>เนื่องจากโดยทั่วไปสันนิษฐานว่าผู้ป่วยจำนวนมากอาจมองข้ามอาการหรือโรคเนื่องจากขาดความรู้ทางการแพทย์<br>แม้แต่ความสงสัยเกี่ยวกับอาการและโรคก็ได้รับการยอมรับและระบุว่าเป็นบวก;<br>อาการที่หายไปโดยสมบูรณ์ให้ระบุว่าเป็นลบ โปรดทราบว่าเราถือว่าเป็นบวกเมื่อผู้ใช้ทานยาที่อาจทำให้หายจากอาการชั่วคราว;

Prompt setting	Language	Prompt
		สำหรับกรณีที่แสดงถึงความคาดหวังหรือกระบวนการ ซึ่งระบุด้วยคำเช่น "ถ้า" "กำลังจะ" "ถ้าเป็น" เป็นต้น ให้ระบุว่าเป็นลบ;<br>หากมีการกล่าวถึงโรคเพียงในฐานะหัวข้อ ไม่ใช่ว่ามีใครเป็นโรคนั้นจริง ทวีตเหล่านี้ให้ระบุว่าเป็นลบ ซึ่งรวมถึงข่าว ทฤษฎีทั่วไปและโฆษณา;<br>หากมีการกล่าวถึงโรคในบริบทของเรื่องตลก ให้ระบุว่าเป็นลบ;<br>อาการนี้ใช้กับมนุษย์เท่านั้น;<br>อาการต้องอยู่ภายใน 24 ชั่วโมงรวมถึงวันนี้;<br>ป้ายกำกับสำหรับอาการที่เกิดขึ้นเมื่อวานขึ้นอยู่กับชนิดของโรคหรืออาการ;<br>อาการในอดีตรวมถึงที่เกิดขึ้นสองวันขึ้นไปก่อนหน้าถือว่าเป็นลบ;<br>การเกิดขึ้นล่าสุดและอาการที่เกิดซ้ำและยังคงมีอยู่ถือว่าเป็นบวก;<br>เราถือว่าเป็นบวกสำหรับอาการของคนใกล้ตัว ไม่ว่าจะอาศัยอยู่ด้วยกันหรือไม่ (เช่น สมาชิกในครอบครัว)<br>เรายังระบุว่าเป็นบวกเมื่อทราบอาการจากการบอกเล่า;<br>สำหรับอาการของผู้คนที่อยู่ในกลุ่มเฉพาะใกล้ตัว (โรงเรียน ชมรม ฯลฯ) เราระบุว่าเป็นบวก กรณีอื่น เช่น อาการของเพื่อนบล็อกให้ระบุว่าเป็นลบเนื่องจากยากที่จะระบุตำแหน่งของพวกเขา;<br>Tweet: {tweet}<br>สำคัญ: คีย์ใน JSON ต้องตรงกับที่ระบุไว้ข้างต้นเท่านั้น: {symptoms_str} ให้ใช้ชื่อเหล่านี้เป็นคีย์อย่างเคร่งครัด<br>ส่งคืนผลลัพธ์เป็นออบเจ็กต์ JSON อย่างเคร่งครัด โดยใช้ชื่ออาการเป็นคีย์และค่าเป็น 0 หรือ 1<br>ตรวจสอบให้แน่ใจว่าผลลัพธ์เป็นรูปแบบ JSON ที่ถูกต้อง<br>ไม่ต้องบอกว่าทวีตนี้เป็นภาษาอะไร ให้ส่งเฉพาะออบเจ็กต์ JSON เท่านั้น<br>ตอบเฉพาะออบเจ็กต์ JSON เท่านั้น ไม่ต้องมีคำอธิบาย ไม่ต้องมี markdown ไม่ต้องมี code fences"""
rule-zero-local	Arabic	"""حدد ما إذا كان مرسل رسالة تويتر هذه يُظهر أعراضًا لكل مما يلي: {symptoms_str}.<br>لكل عرض، أجب فقط بـ 0 أو 1: 0 للسلبي (لا توجد أعراض) و1 للإيجابي (توجد أعراض).<br>يتم تحديد الأعراض بناءً على القواعد التالية:<br>الحالات التي يتم فيها التعبير عن العرض بشكل مباشر، بما في ذلك الأعراض الخفيفة، تُعتبر إيجابية؛<br>يمكن تصنيف العرض على أنه إيجابي حتى مع التعبير غير المباشر عنه؛<br>إذا تم ذكر عرض ما ثم تم إنكاره أو رفضه لاحقًا، فإن هذه المعلومة تُعتبر إيجابية أيضًا؛<br>يُعتبر إيجابيًا إذا كان الشخص أو المستخدم لا يزال متأثرًا بأعراض خفيفة أثناء التعافي. ومع ذلك، إذا اختفت الأعراض تمامًا، فإنها تُعتبر سلبية؛<br>يظل العرض إيجابيًا حتى لو عبّر المستخدم عن عدم اليقين بشأن سببه؛<br>نظرًا لأنه من المفترض عمومًا أن العديد من المرضى قد يتجاهلون الأعراض أو الأمراض بسبب نقص المعرفة الطبية، فإن مجرد الشك في الأعراض والأمراض يُعترف به ويُصنف كإيجابي؛<br>الأعراض التي اختفت تمامًا تُصنف كسلبية. لاحظ أننا نعتبرها ونصنفها كإيجابية عندما يتناول المستخدم دواءً قد يسبب تعافيًا مؤقتًا من عرض ما؛<br>"بالنسبة للحالات التي تعبر عن توقع أو عملية مستقبلية، والمشار إليها بكلمات مثل "إذا"، "سوف"، "إذا كان"، إلخ، يجب تصنيفها كسلبية؛<br>إذا تم ذكر المرض فقط كموضوع وليس كشيء يعاني منه أحد، يجب تصنيف هذه التغريدات كسلبية. ويشمل ذلك الأخبار والنظريات العامة والإعلانات؛<br>إذا تم ذكر المرض في سياق نكتة، يجب تصنيفه كسلبي؛<br>الأعراض تخص البشر فقط؛<br>الأعراض ضمن 24 ساعة بما في ذلك اليوم؛<br>تصنيف الأعراض التي حدثت بالأمس يعتمد على نوع المرض أو العرض؛<br>الأعراض السابقة، بما في ذلك ما حدث قبل يومين أو أكثر، تُعتبر سلبية؛<br>الحدوث الأخير والأعراض المتكررة التي لا تزال مستمرة تُعتبر إيجابية؛<br>نعتبر أعراض الأشخاص المقربين إيجابية بغض النظر عن العيش معًا من عدمه (مثل أفراد الأسرة). كما نصنفها إيجابية عند معرفة العرض عن طريق السماع فقط؛<br>بالنسبة لأعراض الأشخاص المنتمين إلى مجموعة محددة قريبة (مدرسة، نادٍ، إلخ)، نصنفها كإيجابية. الحالات الأخرى، مثل أعراض أصدقاء المدونات، يجب تصنيفها كسلبية لصعوبة تحديد موقعهم؛<br>Tweet: {tweet}<br>مهم: يجب أن تكون مفاتيح JSON مطابقة تمامًا لما ذُكر أعلاه: {symptoms_str}. استخدم هذه الأسماء بدقة كمفاتيح.<br>أعد النتيجة بدقة ككائن JSON، مع الأعراض كمفاتيح والقيم إما 0 أو 1.

Prompt setting	Language	Prompt
		تأكد من أن الناتج بتنسيق JSON صالح.<br>لا تخبرني بلغة التغريدة، فقط قدم كائن JSON.<br>أجب فقط بكائن JSON. بدون شرح، بدون markdown، بدون code fences.""""
rule-zero-local	German	""""Bestimme, ob der Absender dieser Twitter-Nachricht Symptome fuer jedes der folgenden zeigt: {symptoms_str}.<br>Antworte fuer jedes Symptom nur mit 0 oder 1: 0 fuer negativ (keine Symptome) und 1 fuer positiv (Symptome vorhanden).<br>Die Bestimmung der Symptome erfolgt nach folgenden Regeln:<br>Faelle, in denen das Symptom direkt geaeussert wird, einschliesslich milder Symptome, gelten als positiv;<br>Ein Symptom kann auch bei indirekter Aeusserung als positiv gekennzeichnet werden;<br>Wird ein Symptom erwaehnt, aber anschliessend bestritten oder verneint, gilt dies weiterhin als positiv;<br>Es gilt als positiv, wenn jemand oder der Nutzer waehrend der Genesung noch von leichten Symptomen betroffen ist. Sind die Symptome jedoch vollstaendig verschwunden, gilt dies als negativ;<br>Ein Symptom gilt auch dann als positiv, wenn der Nutzer Unsicherheit ueber dessen Ursache aeussert;<br>Da allgemein angenommen wird, dass viele Patienten Symptome oder Krankheiten aufgrund unzureichender medizinischer Kenntnisse uebersehen koennen, wird selbst der Verdacht auf Symptome und Krankheiten als positiv erkannt und gekennzeichnet;<br>Symptome, die vollstaendig verschwunden sind, werden als negativ gekennzeichnet.<br>Beachte, dass wir es als positiv werten, wenn ein Nutzer ein Medikament eingenommen hat, das eine voruebergehende Genesung von einem Symptom bewirken koennte;<br>Bei Faellen, die eine Erwartung oder einen bevorstehenden Vorgang ausdruecken, angezeigt durch Woerter wie "falls", "werde", "wenn es" usw., sollten diese als negativ gekennzeichnet werden;<br>Wird die Krankheit lediglich als Thema erwaehnt und nicht als etwas, das jemand tatsaechlich hat, sollten diese Tweets als negativ gekennzeichnet werden. Dazu zaehlen Nachrichten, allgemeine Theorien und Werbung;<br>Wird die Krankheit im Kontext eines Witzes erwaehnt, sollte dies als negativ gekennzeichnet werden;<br>die Symptome gelten nur fuer Menschen;<br>Symptome liegen innerhalb von 24 Stunden einschliesslich heute;<br>Die Kennzeichnung fuer Symptome, die gestern auftraten, haengt von der Krankheit bzw. dem Symptom ab;<br>Vergangene Symptome, einschliesslich solcher, die vor zwei oder mehr Tagen auftraten, gelten als negativ;<br>Kuerzliches Auftreten und wiederkehrende Symptome, die noch andauern, gelten als positiv;<br>Wir werten Symptome im nahen Umfeld als positiv, unabhaengig davon, ob man zusammenlebt oder nicht (z. B. Familienmitglieder). Wir kennzeichnen auch als positiv, wenn ein Symptom vom Hoerensagen bekannt wurde;<br>Bei Symptomen von Personen, die einer bestimmten nahestehenden Gruppe angehoeren (Schule, Verein usw.), kennzeichnen wir dies als positiv. Andere Faelle, wie Symptome von Blog-Freunden, sollten als negativ gekennzeichnet werden, da ihr Standort schwer zu bestimmen ist;<br>Tweet: {tweet}<br>WICHTIG: Die JSON-Schluessel MUESSEN exakt wie oben aufgefuehrt sein: {symptoms_str}. Verwende diese Namen exakt als Schluessel.<br>Gib das Ergebnis strikt als JSON-Objekt zurueck, mit den Symptomen als Schluessel

Prompt setting	Language	Prompt
		und den Werten 0 oder 1.<br>Stelle sicher, dass die Ausgabe gueltiges JSON-Format ist.<br>Sag mir nicht, in welcher Sprache der Tweet verfasst ist, gib nur das JSON-Objekt an.<br>Antworte NUR mit dem JSON-Objekt. Keine Erklaerung, kein Markdown, keine Code-Fences."""
rule-zero-local	French	Determinez si l'auteur de ce message Twitter presente des symptomes pour chacun des elements suivants : {symptoms_str}.<br>Pour chaque symptome, repondez uniquement par 0 ou 1 : 0 pour negatif (aucun symptome) et 1 pour positif (symptome present).<br>La determination des symptomes se fait selon les regles suivantes :<br>Les cas ou le symptome est exprime directement, y compris les symptomes legers, sont consideres comme positifs ;<br>Un symptome peut etre etiquete positif meme s'il est exprime de maniere indirecte ;<br>Si un symptome est mentionne puis nie ou dementi, cette information est tout de meme consideree comme positive ;<br>Cela est considere comme positif si la personne ou l'utilisateur est encore affecte par ces symptomes legers pendant la convalescence. Cependant, si les symptomes ont completement disparu, cela est considere comme negatif ;<br>Un symptome reste positif meme si l'utilisateur exprime une incertitude quant a sa cause ;<br>Etant donne qu'il est generalement presume que de nombreux patients peuvent negliger des symptomes ou des maladies par manque de connaissances medicales, meme la simple suspicion de symptomes ou de maladies est reconnue et etiquetee comme positive ;<br>Les symptomes ayant completement disparu sont etiquetes comme negatifs. Notez que nous considerons et etiquetons comme positif le cas ou un utilisateur a pris un medicament pouvant entrainer une guerison temporaire d'un symptome ;<br>Pour les cas exprimant une attente ou un processus futur, indiques par des mots comme « si », « je vais », « si c'est », etc., ceux-ci doivent etre etiquetes comme negatifs ;<br>Si la maladie est mentionnee simplement comme sujet et non comme quelque chose que quelqu'un a reellement, ces tweets doivent etre etiquetes comme negatifs. Cela inclut les actualites, les theories generales et les publicites ;<br>Si la maladie est mentionnee dans le contexte d'une blague, cela doit etre etiquete comme negatif ;<br>les symptomes concernent uniquement les humains ;<br>les symptomes doivent etre survenus dans les dernieres 24 heures, y compris aujourd'hui ;<br>L'etiquette pour les symptomes survenus hier depend de la maladie ou du symptome en question ;<br>Les symptomes passes, y compris ceux survenus il y a deux jours ou plus, sont consideres comme negatifs ;<br>Une occurrence recente et un symptome recurrent qui persiste encore sont consideres comme positifs ;<br>Nous considerons comme positifs les symptomes observes chez des proches, qu'ils vivent ensemble ou non (par exemple, des membres de la famille). Nous etiquetons egalement comme positif un symptome rapporte par oui-dire ;<br>Pour les symptomes de personnes appartenant a un groupe specifique proche (ecole, club, etc.), nous les etiquetons comme positifs. D'autres cas, comme les symptomes d'amis de blog, doivent etre etiquetes comme negatifs car il est difficile de determiner leur localisation ;<br>Tweet : {tweet}<br>IMPORTANT : les cles JSON DOIVENT correspondre exactement a celles listees ci-

Prompt setting	Language	Prompt
		dessus : {symptoms_str}. Utilisez ces noms exacts comme cles.<br>Retournez le resultat strictement sous forme d'objet JSON, avec les symptomes comme cles et les valeurs 0 ou 1.<br>Assurez-vous que la sortie est un format JSON valide.<br>Ne me dites pas dans quelle langue est le tweet, fournissez uniquement l'objet JSON.<br>Repondez UNIQUEMENT avec l'objet JSON. Pas d'explication, pas de markdown, pas de code fences."""
rule-zero-local	Khmer	"""កំណត់ថាតើអ្នកផ្ញើសារ Twitter នេះកំពុងបង្ហាញរោគសញ្ញាសម្រាប់នីមួយៗខាងក្រោមឬអត់៖ {symptoms_str}។<br>សម្រាប់រោគសញ្ញានីមួយៗ សូមឆ្លើយតែ 0 ឬ 1 ប៉ុណ្ណោះ៖ 0 សម្រាប់អវិជ្ជមាន (គ្មានរោគសញ្ញា) និង 1 សម្រាប់វិជ្ជមាន (មានរោគសញ្ញា)។<br>ការកំណត់រោគសញ្ញាធ្វើឡើងដោយផ្អែកលើវិធានខាងក្រោម៖<br>ករណីដែលរោគសញ្ញាត្រូវបានបញ្ចេញដោយផ្ទាល់ រួមទាំងរោគសញ្ញាស្រាល ត្រូវបានចាត់ទុកជាវិជ្ជមាន;<br>រោគសញ្ញាអាចត្រូវបានចាត់ថាវិជ្ជមានទោះបីជាបញ្ចេញដោយប្រយោលក៏ដោយ;<br>ប្រសិនបើរោគសញ្ញាមួយត្រូវបានលើកឡើងប៉ុន្តែក្រោយមកត្រូវបានបដិសេធ ព័ត៌មាននេះនៅតែត្រូវបានចាត់ទុកជាវិជ្ជមាន;<br>វាត្រូវបានចាត់ទុកជាវិជ្ជមានប្រសិនបើនរណាម្នាក់ ឬអ្នកប្រើប្រាស់នៅតែរងផលប៉ះពាល់ដោយរោគសញ្ញាស្រាលៗនោះកំឡុងពេលជាសះស្បើយ។ ទោះជាយ៉ាងណា<br>ប្រសិនបើរោគសញ្ញាបានបាត់ទាំងស្រុង វាត្រូវបានចាត់ទុកជាអវិជ្ជមាន;<br>រោគសញ្ញានៅតែជាវិជ្ជមានទោះបីជាអ្នកប្រើប្រាស់បញ្ចេញការមិនច្បាស់លាស់អំពីមូលហេតុរបស់វាក៏ដោយ;<br>ដោយសារជាទូទៅគេសន្មតថាអ្នកជំងឺជាច្រើនអាចមិនកត់សម្គាល់រោគសញ្ញា ឬជំងឺដោយសារខ្វះចំណេះដឹងផ្នែកវេជ្ជសាស្ត្រ សូម្បីតែការសង្ស័យលើរោគសញ្ញា<br>និងជំងឺក៏ត្រូវបានទទួលស្គាល់ និងចាត់ថាវិជ្ជមាន;<br>រោគសញ្ញាដែលបានបាត់ទាំងស្រុងត្រូវបានចាត់ថាអវិជ្ជមាន។<br>សូមចំណាំថាយើងចាត់ទុកជាវិជ្ជមានប្រសិនបើអ្នកប្រើប្រាស់បានលេបថ្នាំដែលអាចធ្វើឱ្យជាសះស្បើយបណ្តោះអាសន្នពីរោគសញ្ញា;<br>សម្រាប់ករណីដែលបង្ហាញពីការរំពឹងទុក ឬដំណើរការនាពេលអនាគត បង្ហាញដោយពាក្យដូចជា "ប្រសិនបើ" "នឹង" "បើសិន" ។ល។ ត្រូវចាត់ថាអវិជ្ជមាន;<br>ប្រសិនបើជំងឺត្រូវបានលើកឡើងគ្រាន់តែជាប្រធានបទ មិនមែនជាអ្វីដែលនរណាម្នាក់កំពុងកើតឡើងនោះទេ សារ tweet ទាំងនេះត្រូវចាត់ថាអវិជ្ជមាន។ រួមទាំងព័ត៌មាន<br>ទូទៅទូទៅ និងការផ្សាយពាណិជ្ជកម្ម;<br>ប្រសិនបើជំងឺត្រូវបានលើកឡើងក្នុងបរិបទនៃការលេងសើច វាត្រូវចាត់ថាអវិជ្ជមាន;<br>រោគសញ្ញាគឺសម្រាប់តែមនុស្សប៉ុណ្ណោះ;<br>រោគសញ្ញាស្ថិតក្នុងរយៈពេល ២៤ ម៉ោង រួមទាំងថ្ងៃនេះ;<br>ស្លាកសម្រាប់រោគសញ្ញាដែលបានកើតឡើងកាលពីម្សិលមិញអាស្រ័យលើប្រភេទជំងឺ ឬរោគសញ្ញា;<br>រោគសញ្ញាកន្លងមក រួមទាំងកើតឡើងកាលពីពីរថ្ងៃ ឬច្រើនថ្ងៃមុន ត្រូវចាត់ថាអវិជ្ជមាន;<br>ការកើតឡើងថ្មីៗ និងរោគសញ្ញាកើតឡើងវិញដែលនៅតែបន្តត្រូវចាត់ថាវិជ្ជមាន;<br>យើងចាត់ទុករោគសញ្ញារបស់អ្នកនៅជិតៗជាវិជ្ជមាន មិនថារស់នៅជាមួយគ្នាឬអត់ (ឧទាហរណ៍ សមាជិកគ្រួសារ)។<br>យើងក៏ចាត់ថាវិជ្ជមានផងដែរនៅពេលដឹងរោគសញ្ញាតាមរយៈការឮគេនិយាយ;<br>ចំពោះរោគសញ្ញារបស់មនុស្សដែលជាកម្មសិទ្ធិរបស់ក្រុមជាក់លាក់នៅជិត (សាលារៀន ក្លឹប ។ល។) យើងចាត់ថាវិជ្ជមាន។ ករណីផ្សេងទៀត ដូចជារោគសញ្ញារបស់មិត្តបុក<br>ត្រូវចាត់ថាអវិជ្ជមាន ព្រោះពិបាកកំណត់ទីតាំងរបស់ពួកគេ;<br>Tweet: {tweet}<br>សំខាន់៖ សោ JSON ត្រូវតែដូចគ្នាបេះបិទនឹងអ្វីដែលបានរាយខាងលើ៖ {symptoms_str}។ សូមប្រើឈ្មោះទាំងនេះឲ្យបានត្រឹមត្រូវជាសោ។<br>សូមបញ្ជូនលទ្ធផលយ៉ាងតឹងរឹងជា JSON object ដោយប្រើឈ្មោះរោគសញ្ញាជាសោ និងតម្លៃជា 0 ឬ 1។<br>សូមប្រាកដថាលទ្ធផលមានទម្រង់ JSON ត្រឹមត្រូវ។<br>មិនចាំបាច់ប្រាប់ខ្ញុំថាសារ tweet នេះជាភាសាអ្វីទេ គ្រាន់តែផ្តល់ JSON object។<br>សូមឆ្លើយតែជា JSON object ប៉ុណ្ណោះ។ គ្មានការពន្យល់ គ្មាន markdown គ្មាន code fences។"""
rule-zero-local	Lao	"""ກຳນົດວ່າຜູ້ສົ່ງຂໍ້ຄວາມ Twitter ນີ້ສະແດງອາການສຳລັບແຕ່ລະຢ່າງຕໍ່ໄປນີ້ຫຼືບໍ່: {symptoms_str}.

Prompt setting	Language	Prompt
		ສຳລັບແຕ່ລະອາການ ໃຫ້ຕອບພຽງແຕ່ 0 ຫຼື 1: 0 ສຳລັບລົບ (ບໍ່ມີອາການ) ແລະ 1<br>ສຳລັບບວກ (ມີອາການ).<br>ການກຳນົດອາການແມ່ນອີງໃສ່ກົດລະບຽບຕໍ່ໄປນີ້:<br>ກໍລະນີທີ່ອາການຖືກສະແດງອອກໂດຍກົງ ລວມທັງອາການອ່ອນໆ ຖືວ່າເປັນບວກ;<br>ອາການສາມາດຖືກລະບຸເປັນບວກໄດ້ເຖິງແມ່ນວ່າຈະສະແດງອອກທາງອ້ອມ;<br>ຖ້າອາການຖືກກ່າວເຖິງແຕ່ພາຍຫຼັງຖືກປະຕິເສດ ຂໍ້ມູນນີ້ຍັງຖືວ່າເປັນບວກ;<br>ຖືວ່າເປັນບວກຖ້າຄົນຫຼືຜູ້ໃຊ້ຍັງໄດ້ຮັບຜົນກະທົບຈາກອາການອ່ອນໆເຫຼົ່ານັ້ນໃນຊ່ວງ<br>ຟື້ນຟູ. ແນວໃດກໍ່ຕາມ ຖ້າອາການໄດ້ຫາຍໄປໝົດແລ້ວ ຖືວ່າເປັນລົບ;<br>ອາການຍັງຄົງເປັນບວກເຖິງແມ່ນວ່າຜູ້ໃຊ້ຈະສະແດງຄວາມບໍ່ແນ່ໃຈກ່ຽວກັບສາເຫດ;<br>ເນື່ອງຈາກໂດຍທົ່ວໄປສົມມຸດວ່າຄົນເຈັບຫຼາຍຄົນອາດຈະລືມອາການ<br>ຫຼືພະຍາດເນື່ອງຈາກຂາດຄວາມຮູ້ທາງການແພດ<br>ແມ້ແຕ່ຄວາມສົງໄສກ່ຽວກັບອາການ ແລະພະຍາດກໍ່ຖືກຮັບຮູ້ ແລະລະບຸເປັນບວກ;<br>ອາການທີ່ຫາຍໄປໝົດແລ້ວຖືກລະບຸເປັນລົບ.<br>ໃຫ້ສັງເກດວ່າພວກເຮົາຖືວ່າເປັນບວກເມື່ອຜູ້ໃຊ້ກິນຢາທີ່ອາດເຮັດໃຫ້ຟື້ນຕົວຊົ່ວຄາ<br>ວຈາກອາການ;<br>ສຳລັບກໍລະນີທີ່ສະແດງຄວາມຄາດຫວັງ ຫຼືຂະບວນການໃນອະນາຄົດ<br>ຊີ້ບອກດ້ວຍຄຳເຊັ່ນ "ຖ້າ" "ຈະ" "ຖ້າວ່າ" ແລະອື່ນໆ ໃຫ້ລະບຸເປັນລົບ;<br>ຖ້າພະຍາດຖືກກ່າວເຖິງພຽງແຕ່ເປັນຫົວຂໍ້ ບໍ່ແມ່ນສິ່ງທີ່ໃຜຄົນໜຶ່ງກຳລັງເປັນ tweet<br>ເຫຼົ່ານີ້ຄວນຖືກລະບຸເປັນລົບ. ນີ້ລວມມີຂ່າວ, ທິດສະດີທົ່ວໄປ, ແລະການໂຄສະນາ;<br>ຖ້າພະຍາດຖືກກ່າວເຖິງໃນສະພາບການເລື່ອງຕະຫຼົກ ໃຫ້ລະບຸເປັນລົບ;<br>ອາການແມ່ນສຳລັບມະນຸດເທົ່ານັ້ນ;<br>ອາການແມ່ນຢູ່ພາຍໃນ 24 ຊົ່ວໂມງ ລວມທັງມື້ນີ້;<br>ປ້າຍກຳກັບສຳລັບອາການທີ່ເກີດຂຶ້ນມື້ວານແມ່ນຂຶ້ນກັບປະເພດຂອງພະຍາດ<br>ຫຼືອາການ;<br>ອາການໃນອະດີດ ລວມທັງທີ່ເກີດຂຶ້ນສອງມື້ ຫຼືຫຼາຍກວ່ານັ້ນກ່ອນໜ້ານີ້<br>ຖືວ່າເປັນລົບ;<br>ການເກີດຂຶ້ນຫາກໆ ແລະອາການທີ່ເກີດຂຶ້ນຄືນໃໝ່ ແລະຍັງດຳເນີນຢູ່<br>ຖືວ່າເປັນບວກ;<br>ພວກເຮົາຖືວ່າອາການຂອງຄົນໃກ້ຄຽງເປັນບວກ ບໍ່ວ່າຈະຢູ່ຮ່ວມກັນ ຫຼືບໍ່ (ເຊັ່ນ<br>ສະມາຊິກຄອບຄົວ).<br>ພວກເຮົາຍັງລະບຸເປັນບວກເມື່ອຮູ້ອາການຈາກການໄດ້ຍິນຄົນອື່ນເວົ້າ;<br>ສຳລັບອາການຂອງຄົນທີ່ຢູ່ໃນກຸ່ມສະເພາະໃກ້ຄຽງ (ໂຮງຮຽນ, ຊົມຮົມ, ແລະອື່ນໆ),<br>ພວກເຮົາລະບຸເປັນບວກ. ກໍລະນີອື່ນໆ ເຊັ່ນ ອາການຂອງໝູ່ blog ຄວນລະບຸເປັນລົບ<br>ເພາະຍາກທີ່ຈະກຳນົດສະຖານທີ່ຂອງເຂົາເຈົ້າ;<br>Tweet: {tweet}<br>ສຳຄັນ: ກະແຈ JSON ຕ້ອງກົງກັບທີ່ລະບຸໄວ້ຂ້າງເທິງນີ້ຢ່າງແທ້ຈິງ: {symptoms_str}.<br>ກະລຸນາໃຊ້ຊື່ເຫຼົ່ານີ້ຢ່າງຖືກຕ້ອງເປັນກະແຈ.<br>ກະລຸນາສົ່ງຄືນຜົນລັບຢ່າງເຂັ້ມງວດເປັນ JSON object ໂດຍໃຊ້ຊື່ອາການເປັນກະແຈ<br>ແລະຄ່າເປັນ 0 ຫຼື 1.<br>ໃຫ້ແນ່ໃຈວ່າຜົນອອກມາເປັນຮູບແບບ JSON ທີ່ຖືກຕ້ອງ.<br>ບໍ່ຕ້ອງບອກຂ້ອຍວ່າ tweet ນີ້ເປັນພາສາຫຍັງ ໃຫ້ສະໜອງແຕ່ JSON object.<br>ຕອບພຽງແຕ່ JSON object ເທົ່ານັ້ນ. ບໍ່ມີຄຳອະທິບາຍ, ບໍ່ມີ markdown, ບໍ່ມີ code<br>fences."""
rule-zero-local	Burmese	"""ဤ Twitter စာတိုကို ပေးပို့သူသည် အောက်ပါလက္ခဏာများအနက် တစ်ခုစီအတွက်<br>လက္ခဏာများ ပြသနေခြင်း ရှိမရှိ ဆုံးဖြတ်ပါ: {symptoms_str}။<br>လက္ခဏာတစ်ခုစီအတွက် 0 သို့မဟုတ် 1 ဖြင့်သာ ဖြေဆိုပါ- 0 သည် အနှုတ်<br>(လက္ခဏာမရှိ)၊ 1 သည် အပေါင်း (လက္ခဏာရှိ)။

Prompt setting	Language	Prompt
		လက္ခဏာများကို အောက်ပါစည်းမျဉ်းများအပေါ် အခြေခံ၍ ဆုံးဖြတ်ပါ-<br>လက္ခဏာအနည်းငယ်အပါအဝင် တိုက်ရိုက်ဖော်ပြထားသော အခြေအနေများကို အပေါင်းဟု သတ်မှတ်သည်;<br>သွယ်ဝိုက်၍ဖော်ပြထားသော်လည်း လက္ခဏာကို အပေါင်းဟု သတ်မှတ်နိုင်သည်;<br>လက္ခဏာတစ်ခုကို ဖော်ပြပြီးနောက် ငြင်းဆိုသည့်တိုင် ထိုအချက်အလက်ကို အပေါင်းဟု ယူဆသည်;<br>ပြန်လည်ကောင်းမွန်လာစဉ် ယင်းအနည်းငယ်သောလက္ခဏာများက ဆက်လက်ခံစားနေရသေးလျှင် အပေါင်းဟု သတ်မှတ်သည်။ သို့သော် လက္ခဏာများ လုံးဝပျောက်ကွယ်သွားလျှင် အနှုတ်ဟု ယူဆသည်;<br>အကြောင်းရင်းနှင့်ပတ်သက်၍ မသေချာမှုကို ဖော်ပြထားသော်လည်း လက္ခဏာသည် အပေါင်းဖြစ်နေဆဲဖြစ်သည်;<br>ဆေးပညာဗဟုသုတ မလုံလောက်ခြင်းကြောင့် လူနာအများအပြားသည် လက္ခဏာ သို့မဟုတ် ရောဂါများကို လျစ်လျူရှုမိနိုင်သည်ဟု ယေဘုယျအားဖြင့် ယူဆထားသောကြောင့် လက္ခဏာနှင့် ရောဂါများအား သံသယရှိရုံဖြင့်ပင် အသိအမှတ်ပြု၍ အပေါင်းဟု သတ်မှတ်သည်;<br>လုံးဝပျောက်ကွယ်သွားသော လက္ခဏာများကို အနှုတ်ဟု သတ်မှတ်သည်။<br>အသုံးပြုသူသည် လက္ခဏာတစ်ခုမှ ယာယီပြန်ကောင်းလာစေနိုင်သော ဆေးဝါးကို သောက်ခဲ့ပါက ၎င်းကို အပေါင်းဟု ယူဆ၍ သတ်မှတ်ကြောင်း သတိပြုပါ;<br>"ဆိုရင်"၊ "သွားတော့မယ်"၊ "ဆိုရင်တော့" စသည့်စကားလုံးများဖြင့် ဖော်ပြထားသည့် မျှော်လင့်ချက် သို့မဟုတ် ဖြစ်စဉ်ကို ဖော်ပြသည့် အခြေအနေများကို အနှုတ်ဟု သတ်မှတ်ရမည်;<br>ရောဂါကို တစ်စုံတစ်ယောက် ခံစားနေရသောအရာအဖြစ်မဟုတ်ဘဲ ခေါင်းစဉ်တစ်ခုအနေဖြင့်သာ ဖော်ပြထားလျှင် ထိုတွစ်များကို အနှုတ်ဟု သတ်မှတ်ရမည်။ ၎င်းတွင် သတင်း၊ ယေဘုယျသီအိုရီများနှင့် ကြော်ငြာများ ပါဝင်သည်;<br>ရောဂါကို ဟာသအနေဖြင့် ဖော်ပြထားလျှင် အနှုတ်ဟု သတ်မှတ်ရမည်;<br>လက္ခဏာများသည် လူသားများအတွက်သာ သက်ဆိုင်သည်;<br>လက္ခဏာများသည် ယနေ့အပါအဝင် ၂၄ နာရီအတွင်း ဖြစ်ပေါ်သည်;<br>မနေ့ကဖြစ်ပွားသော လက္ခဏာများအတွက် တံဆိပ်သည် ရောဂါ သို့မဟုတ် လက္ခဏာအမျိုးအစားပေါ်မူတည်သည်;<br>နှစ်ရက် သို့မဟုတ် ထို့ထက်ပို၍ အတိတ်ကဖြစ်ခဲ့သော လက္ခဏာများကို အနှုတ်ဟု ယူဆသည်;<br>မကြာသေးမီကဖြစ်ပွားပြီး ယခုတိုင်ဆက်လက်ရှိနေသေးသော ထပ်ခါထပ်ခါဖြစ်ပွားသည့် လက္ခဏာများကို အပေါင်းဟု ယူဆသည်;

Prompt setting	Language	Prompt
		အနီးတဝိုက်ရှိလူများ (ဥပမာ မိသားစုဝင်များ) အတူနေထိုင်သည်ဖြစ်စေ မနေသည်ဖြစ်စေ ၎င်းတို့၏လက္ခဏာများကို အပေါင်းဟု ယူဆသည်။ ကြားဖူးမှုမှသိရသော လက္ခဏာများကိုလည်း အပေါင်းဟု သတ်မှတ်သည်;<br>အနီးတဝိုက်ရှိ သတ်မှတ်ထားသောအုပ်စု (ကျောင်း၊ ကလပ် စသည်) ဝင်များ၏ လက္ခဏာများကို အပေါင်းဟု သတ်မှတ်သည်။ blog သူငယ်ချင်းများ၏ လက္ခဏာကဲ့သို့ တည်နေရာသိရန်ခက်ခဲသော အခြားအခြေအနေများကို အနှုတ်ဟု သတ်မှတ်ရမည်;<br>Tweet: {tweet}<br>အရေးကြီးသည်- JSON key များသည် အထက်တွင်ဖော်ပြထားသည့်အတိုင်း အတိအကျဖြစ်ရမည်- {symptoms_str}။ ဤအမည်များကို key အဖြစ် အတိအကျအသုံးပြုပါ။<br>ရလဒ်ကို လက္ခဏာများကို key အဖြစ်၊ တန်ဖိုးများကို 0 သို့မဟုတ် 1 အဖြစ်ဖြင့် JSON object အနေဖြင့် တိကျစွာ ပြန်ပေးပါ။<br>output သည် မှန်ကန်သော JSON format ဖြစ်ကြောင်း သေချာပါစေ။<br>tweet သည် မည်သည့်ဘာသာစကားဖြစ်သည်ကို ပြောပြရန် မလိုအပ်ပါ၊ JSON object ကိုသာ ပေးပါ။<br>JSON object ကိုသာ ဖြေကြားပါ။ ရှင်းလင်းချက် မလိုအပ်ပါ၊ markdown မလိုအပ်ပါ၊ code fences မလိုအပ်ပါ။"""
rule-fewshot-english	English	"Determine if the sender of this Twitter message is exhibiting symptoms for each of the following: {symptoms_str}.",<br>"For each symptom, only answer either 0 or 1 for negative (no symptoms) or positive (has symptoms) respectively.",<br>"Below are the rules used to determine symptoms. Each rule is immediately followed by ONE example showing exactly how that rule applies."<br><br>{<br>"rule": "Cases where the symptom is expressed directly including mild symptoms are considered positive.",<br>"tweet": "It's a mild headache, it'll be fine.", # 1622en<br>"label": {"Influenza": 0, "Diarrhea": 0, "Hayfever": 0, "Cough": 0, "Headache": 1, "Fever": 0, "Runnynose": 0, "Cold": 0},<br>},<br>{<br>"rule": "A symptom can be labeled positive with indirect expressions of a symptom.",<br>"tweet": "I just got a prescription for allergy medicine.", # 256en<br>"label": {"Influenza": 0, "Diarrhea": 0, "Hayfever": 1, "Cough": 0, "Headache": 0, "Fever": 0, "Runnynose": 1, "Cold": 0},<br>},<br>{<br>"rule": "If a symptom is mentioned but then also dismissed or denied, this information is regarded as positive.",<br>"tweet": "I have a fever, but it's no big deal.", # 836en<br>"label": {"Influenza": 0, "Diarrhea": 0, "Hayfever": 0, "Cough": 0, "Headache": 0, "Fever": 1, "Runnynose": 0, "Cold": 0},

Prompt setting	Language	Prompt
		},<br>{<br>"rule": "It is considered positive if someone or the user is still affected with such mild symptoms during recovery. However, if the symptoms are completely gone, it is considered negative.",<br>"tweet": "I'm happy, my stuffy nose is finally better.", # 1640en<br>"label": {"Influenza": 0, "Diarrhea": 0, "Hayfever": 0, "Cough": 0, "Headache": 0, "Fever": 0, "Runnynose": 1, "Cold": 0},<br>},<br>{<br>"rule": "A symptom is positive even if the user expresses uncertainty regarding its cause.",<br>"tweet": "I've had a bad headache since this morning. I wonder if that's because I drank too much yesterday.", # 24en<br>"label": {"Influenza": 0, "Diarrhea": 0, "Hayfever": 0, "Cough": 0, "Headache": 1, "Fever": 0, "Runnynose": 0, "Cold": 0},<br>},<br>{<br>"rule": "Since it is generally presumed that many patients may overlook symptoms or diseases due to insufficient medical knowledge, even suspicion of symptoms and diseases are recognized and labeled positive.",<br>"tweet": "I feel so groggy. I might have a cold.", # 1037en<br>"label": {"Influenza": 0, "Diarrhea": 0, "Hayfever": 0, "Cough": 0, "Headache": 0, "Fever": 0, "Runnynose": 0, "Cold": 1},<br>},<br>{<br>"rule": "Symptoms that disappeared completely are recognized and labeled negative. Note that we regarded and labeled positive when a user took medicine that could cause temporary recovery from a symptom.",<br>"tweet": "I took the medicine prescribed to me by the doctor, and my allergy symptoms got better.", # 1770en<br>"label": {"Influenza": 0, "Diarrhea": 0, "Hayfever": 1, "Cough": 0, "Headache": 0, "Fever": 0, "Runnynose": 1, "Cold": 0},<br>},<br>{<br>"rule": 'For cases that express expectation or process, indicated with words such as "if," "going," "if it is," etc., these should be labeled as negative.',<br>"tweet": "If you have a cold, please get plenty of rest.", # 354en<br>"label": {"Influenza": 0, "Diarrhea": 0, "Hayfever": 0, "Cough": 0, "Headache": 0, "Fever": 0, "Runnynose": 0, "Cold": 0},<br>},<br>{<br>"rule": "If the disease is mentioned merely as a topic rather than someone having it, these tweets should be labeled as negative. These include news, general theories, and advertisements.",<br>"tweet": "I heard that in China, someone died of a new type of flu resulting from the bird flu. That got me wondering if there is any preparation like some kit to check to see if I have the flu or not. They talk about the flu a lot on TV, but I have no idea how they manage the outbreak.", # 295en<br>"label": {"Influenza": 0, "Diarrhea": 0, "Hayfever": 0, "Cough": 0, "Headache": 0, "Fever": 0, "Runnynose": 0, "Cold": 0},<br>},<br>{

Prompt setting	Language	Prompt
		"rule": "If the disease is mentioned in the context of a joke, these should be labeled as negative.",<br>"tweet": "Every time I think about contracts, I get a headache.", # 1083en<br>"label": {"Influenza": 0, "Diarrhea": 0, "Hayfever": 0, "Cough": 0, "Headache": 0, "Fever": 0, "Runnynose": 0, "Cold": 0},<br>},<br>{<br>"rule": "The symptoms are only for humans.",<br>"tweet": "The dog's nose was running lol", # 1388en<br>"label": {"Influenza": 0, "Diarrhea": 0, "Hayfever": 0, "Cough": 0, "Headache": 0, "Fever": 0, "Runnynose": 0, "Cold": 0},<br>},<br>{<br>"rule": "Symptoms are within 24 hours including today.",<br>"tweet": "It's been cold lately and I haven't been in great shape. Today I got diarrhea.", # 218en<br>"label": {"Influenza": 0, "Diarrhea": 1, "Hayfever": 0, "Cough": 0, "Headache": 0, "Fever": 0, "Runnynose": 0, "Cold": 0},<br>},<br>{<br>"rule": "The label for symptoms that occurred yesterday are dependent on the disease or symptom.",<br>"tweet": "I have had a fever since yesterday and my throat is swollen. I can sleep as long as I want. I'm not going to work tomorrow either.", # 212en<br>"label": {"Influenza": 0, "Diarrhea": 0, "Hayfever": 0, "Cough": 1, "Headache": 0, "Fever": 1, "Runnynose": 0, "Cold": 0},<br>},<br>{<br>"rule": "Past symptoms including two or more days ago considered as negative.",<br>"tweet": "Last week, my brother got the flu, but now he's doing fine.", # 552en<br>"label": {"Influenza": 0, "Diarrhea": 0, "Hayfever": 0, "Cough": 0, "Headache": 0, "Fever": 0, "Runnynose": 0, "Cold": 0},<br>},<br>{<br>"rule": "Recent occurrence and recurring symptom that still persists considered as positive.",<br>"tweet": "It's been a while since I've had allergy symptoms.", # 2en<br>"label": {"Influenza": 0, "Diarrhea": 0, "Hayfever": 1, "Cough": 0, "Headache": 0, "Fever": 0, "Runnynose": 1, "Cold": 0},<br>},<br>{<br>"rule": "We regarded as a symptom in the vicinity and labeled positive regardless of living together or not (i.e. family members). We also labeled positive when a symptom was observed from hearsay.",<br>"tweet": "My brother seems to have a fever and keeps sniffling. Cut it out.", # 1254en<br>"label": {"Influenza": 0, "Diarrhea": 0, "Hayfever": 0, "Cough": 0, "Headache": 0, "Fever": 1, "Runnynose": 1, "Cold": 0},<br>},<br>{<br>"rule": "As for symptoms of people belonging to a specified group in the vicinity (school, club, etc.), we labeled them positive. Other cases, like symptoms belonging to blog friends, should be labeled as negative since it is difficult to determine their location.",

Prompt setting	Language	Prompt
		"tweet": "I have to be careful since the flu is going around at my club.", # 1368en<br>"label": {"Influenza": 1, "Diarrhea": 0, "Hayfever": 0, "Cough": 0, "Headache": 0, "Fever": 1, "Runnynose": 0, "Cold": 0},<br>},<br><br>IMPORTANT: JSON keys MUST be exactly as listed above: {symptoms_str}.<br>Return the result strictly as a JSON object with the symptoms as keys and the values as either 0 or 1.<br>Ensure that the output is valid JSON format.<br>Do not tell me what language the tweet is in, just provide the JSON object.<br>Respond with ONLY the JSON object. No explanation, no markdown, no code fences."""
basic-zero-english	English	Determine if the sender of this Twitter message is exhibiting symptoms for each of the following: {symptoms_str}.<br>For each symptom, answer only 0 (no symptom) or 1 (has symptom).<br>Tweet: {tweet}<br>IMPORTANT: JSON keys MUST be exactly as listed above: {symptoms_str}.<br>Return the result strictly as a JSON object with the symptoms as keys and the values as either 0 or 1.<br>Respond with ONLY the JSON object. No explanation, no markdown, no code fences."""
basic-zero-local	Indonesian	"""Tentukan apakah pengirim pesan Twitter ini menunjukkan gejala untuk masing-masing berikut: {symptoms_str}.<br>Untuk setiap gejala, jawab hanya 0 (tidak ada gejala) atau 1 (ada gejala).<br>Tweet: {tweet}<br>PENTING: kunci JSON HARUS persis seperti di atas: {symptoms_str}.<br>Kembalikan hasilnya secara ketat sebagai objek JSON dengan gejala sebagai kunci dan nilai 0 atau 1.<br>Jawab HANYA dengan objek JSON. Tanpa penjelasan, tanpa markdown, tanpa code fences."""
basic-zero-local	Malay	"""Tentukan sama ada penghantar mesej Twitter ini menunjukkan gejala bagi setiap yang berikut: {symptoms_str}.<br>Bagi setiap gejala, jawab hanya 0 (tiada gejala) atau 1 (ada gejala).<br>Tweet: {tweet}<br>PENTING: kunci JSON MESTI sama persis seperti di atas: {symptoms_str}.<br>Kembalikan hasil secara ketat sebagai objek JSON dengan gejala sebagai kunci dan nilai 0 atau 1.<br>Jawab HANYA dengan objek JSON. Tiada penjelasan, tiada markdown, tiada code fences."""
basic-zero-local	Filipino	"""Tukuyin kung ang nagpadala ng mensaheng Twitter na ito ay nagpapakita ng sintomas para sa bawat isa sa mga sumusunod: {symptoms_str}.<br>Para sa bawat sintomas, sumagot lamang ng 0 (walang sintomas) o 1 (may sintomas).<br>Tweet: {tweet}<br>MAHALAGA: ang mga JSON key ay DAPAT eksaktong katulad ng nakalista sa itaas: {symptoms_str}.<br>Ibalik ang resulta nang mahigpit bilang JSON object kung saan ang mga sintomas ang keys at ang values ay 0 o 1.<br>Sumagot LAMANG ng JSON object. Walang paliwanag, walang markdown, walang code fences."""
basic-zero-local	Japanese	"""以下のそれぞれの症状について、この Twitter メッセージの発信者が症状を示しているかどうかを判定してください: {symptoms_str}。

Prompt setting	Language	Prompt
		各症状について、0（症状なし）または 1（症状あり）のみで答えてください。<br>Tweet: {tweet}<br>重要: JSON のキーは必ず上記の通りにしてください: {symptoms_str}。<br>症状をキーとし、値を 0 または 1 とした JSON オブジェクトとして、結果を厳密に返してください。<br>JSON オブジェクトのみで回答してください。説明、マークダウン、コードフェンスは不要です。"""
basic-zero-local	Thailand	"""พิจารณาว่าผู้ส่งข้อความ Twitter นี้แสดงอาการของแต่ละอาการต่อไปนี้หรือไม่: {symptoms_str}<br>สำหรับแต่ละอาการ ให้ตอบเพียง 0 (ไม่มีอาการ) หรือ 1 (มีอาการ)<br>Tweet: {tweet}<br>สำคัญ: คีย์ใน JSON ต้องตรงกับที่ระบุไว้ข้างต้นเท่านั้น: {symptoms_str}<br>ส่งคืนผลลัพธ์เป็นออบเจ็กต์ JSON อย่างเคร่งครัด โดยใช้ชื่ออาการเป็นคีย์และค่าเป็น 0 หรือ 1<br>ตอบเฉพาะออบเจ็กต์ JSON เท่านั้น ไม่ต้องมีคำอธิบาย ไม่ต้องมี markdown ไม่ต้องมี code fences"""
basic-zero-local	Arabic	"""حدد ما إذا كان مرسل رسالة تويتر هذه يُظهر أعراضًا لكل مما يلي: {symptoms_str}.<br>لكل عرض، أجب فقط بـ 0 (لا يوجد عرض) أو 1 (يوجد عرض).<br>Tweet: {tweet}<br>مهم: يجب أن تكون مفاتيح JSON مطابقة تمامًا لما ذُكر أعلاه: {symptoms_str}.<br>أعد النتيجة بدقة ككائن JSON، مع الأعراض كمفاتيح والقيم إما 0 أو 1.<br>أجب فقط بكائن JSON. بدون شرح، بدون markdown، بدون code fences."""
basic-zero-local	German	"""Bestimme, ob der Absender dieser Twitter-Nachricht Symptome fuer jedes der folgenden zeigt: {symptoms_str}.<br>Antworte fuer jedes Symptom nur mit 0 (kein Symptom) oder 1 (Symptom vorhanden).<br>Tweet: {tweet}<br>WICHTIG: Die JSON-Schluessel MUESSEN exakt wie oben aufgefuehrt sein: {symptoms_str}.<br>Gib das Ergebnis strikt als JSON-Objekt zurueck, mit den Symptomen als Schluessel und den Werten 0 oder 1.<br>Antworte NUR mit dem JSON-Objekt. Keine Erklaerung, kein Markdown, keine Code-Fences."""
basic-zero-local	French	"""Determinez si l'auteur de ce message Twitter presente des symptomes pour chacun des elements suivants : {symptoms_str}.<br>Pour chaque symptome, repondez uniquement par 0 (aucun symptome) ou 1 (symptome present).<br>Tweet : {tweet}<br>IMPORTANT : les cles JSON DOIVENT correspondre exactement a celles listees ci-dessus : {symptoms_str}.<br>Retournez le resultat strictement sous forme d'objet JSON, avec les symptomes comme cles et les valeurs 0 ou 1.<br>Repondez UNIQUEMENT avec l'objet JSON. Pas d'explication, pas de markdown, pas de code fences."""
basic-zero-local	Khmer	"""កំណត់ថាតើអ្នកផ្ញើសារ Twitter នេះកំពុងបង្ហាញរោគសញ្ញាសម្រាប់នីមួយៗខាងក្រោមឬអត់៖ {symptoms_str}។<br>សម្រាប់រោគសញ្ញានីមួយៗ សូមឆ្លើយតែ 0 (គ្មានរោគសញ្ញា) ឬ 1 (មានរោគសញ្ញា) ប៉ុណ្ណោះ។<br>Tweet: {tweet}<br>សំខាន់៖ សោ JSON ត្រូវតែដូចគ្នាបេះបិទនឹងអ្វីដែលបានរាយខាងលើ៖ {symptoms_str}។<br>សូមបញ្ជូនលទ្ធផលយ៉ាងតឹងរឹងជា JSON object ដោយប្រើឈ្មោះរោគសញ្ញាជាសោ និងតម្លៃជា 0 ឬ 1។<br>សូមឆ្លើយតែជា JSON object ប៉ុណ្ណោះ។ គ្មានការពន្យល់ គ្មាន markdown គ្មាន code fences។"""

Prompt setting	Language	Prompt
basic-zero-local	Lao	"""ກຳນົດວ່າຜູ້ສົ່ງຂໍ້ຄວາມ Twitter ນີ້ສະແດງອາການສຳລັບແຕ່ລະຢ່າງຕໍ່ໄປນີ້ຫຼືບໍ່: {symptoms_str}.<br>ສຳລັບແຕ່ລະອາການ ໃຫ້ຕອບພຽງແຕ່ 0 (ບໍ່ມີອາການ) ຫຼື 1 (ມີອາການ).<br>Tweet: {tweet}<br>ສຳຄັນ: ກະແຈ JSON ຕ້ອງກົງກັບທີ່ລະບຸໄວ້ຂ້າງເທິງນີ້ຢ່າງແທ້ຈິງ: {symptoms_str}.<br>ກະລຸນາສົ່ງຄືນຜົນລັບຢ່າງເຂັ້ມງວດເປັນ JSON object ໂດຍໃຊ້ຊື່ອາການເປັນກະແຈ ແລະຄ່າເປັນ 0 ຫຼື 1.<br>ຕອບພຽງແຕ່ JSON object ເທົ່ານັ້ນ. ບໍ່ມີຄຳອະທິບາຍ, ບໍ່ມີ markdown, ບໍ່ມີ code fences."""
basic-zero-local	Burmese	"""ဤ Twitter စာတိုကို ပေးပို့သူသည် အောက်ပါလက္ခဏာများအနက် တစ်ခုစီအတွက် လက္ခဏာများ ပြသနေခြင်း ရှိမရှိ ဆုံးဖြတ်ပါ: {symptoms_str}။<br>လက္ခဏာတစ်ခုစီအတွက် 0 (လက္ခဏာမရှိ) သို့မဟုတ် 1 (လက္ခဏာရှိ) ဖြင့်သာ ဖြေဆိုပါ။<br>Tweet: {tweet}<br>အရေးကြီးသည်- JSON key များသည် အထက်တွင်ဖော်ပြထားသည့်အတိုင်း အတိအကျဖြစ်ရမည်- {symptoms_str}။<br>ရလဒ်ကို လက္ခဏာများကို key အဖြစ်၊ တန်ဖိုးများကို 0 သို့မဟုတ် 1 အဖြစ်ဖြင့် JSON object အနေဖြင့် တိကျစွာ ပြန်ပေးပါ။<br>JSON object ကိုသာ ဖြေကြားပါ။ ရှင်းလင်းချက် မလိုအပ်ပါ၊ markdown မလိုအပ်ပါ၊ code fences မလိုအပ်ပါ။"""
