# Supplementary material for: Multilingual Disparities in Large Language Model–Based Symptom Detection for Global Disease Surveillance: Evaluation Study
Source: JMIR AI. 2026 Oct 9;5:e103321. doi: 10.2196/103321 (PMC13652523; doi:10.2196/103321)
Supplement: Supplementary file 2 [file ai-v5-e103321-s002.pdf]

Multimedia Appendix 2: Error Analysis

Table S3. Examples of misclassified tweets along with their error classifications. Italic words in the tweet column indicate keywords related to the symptoms.

Case id	Language	Tweet	True Labels	Predicted Labels	Error Classification
2512	en	I've got the *flu*, can't shake hands	FLU, FEV	FLU	Explicitly mentioned symptom
	ms	Saya ada *demam selesema*, tak boleh bersalam	FLU, FEV	FLU, FEV, RUN	Cross-lingual variation + Symptom overgeneralization
2045	en	I think I have a *cold*, I feel out of it.	CLD	CLD	-
	fil	May *sipon* yata ako, pakiramdam ko wala ako sa aking sarili.	CLD	RUN	Cross-lingual variation
	id	Tampaknya aku *pilek*, aku merasa nggak enak badan.	CLD	RUN, CLD	Cross-lingual variation
2119	en	Ugh, I have a *headache* and chills because of this *cold*, I'm so out of it	CLD, HEA	CLD, HEA, FEV	Symptom overgeneralizaion
	ms	Uhh, saya *sakit kepala* dan menggigil disebabkan *selesema* ni. Tak tahan saya.	CLD, HEA	FLU, HEA, FEV	Cross-lingual variation + Symptom overgeneralization
2165	en	I thought I had the *flu* so I went to the doctor, but I got tested and I was wrong.	No positive symptoms	No positive symptoms	-
	ar	اعتقدت أنني مصاب بالإنفلونزا فذهبت إلى الطبيب، لكنني أجريت الاختبار وكنت مخطئًا.	No positive symptoms	No positive symptoms	-
	de	Ich dachte, ich hätte die *Grippe*, also habe ich mich beim Arzt testen lassen und lag falsch.	No positive symptoms	FLU	context misinterpretation
	fr	Je pensais avoir la *grippe* alors je suis allé(e) chez le médecin où j'ai passé un test mais j'avais tort.	No positive symptoms	FLU	context misinterpretation

Table S4. Distribution of the number of error tweets assigned to each primary error category across rule-based prompting strategies.

Error Category	Number of error tweet		
	Rule-zero-en	Rule-fewshot-en	Rule-zero-local
Context misinterpretation	61	67	63
Cross-lingual variation	128	117	127
Explicitly mentioned symptoms	86	64	81
Symptom overgeneralization	18	18	18
Unclear	3	4	4

Table S5. Distribution of error tweets across error categories using multilabel classification for each rule-based prompting strategy. Each error tweet could be assigned to more than one error category; therefore, the category counts may exceed the total number of error tweets.

Error Category	Number of error tweet		
	Rule-zero-en	Rule-fewshot-en	Rule-zero-local
Context misinterpretation	133	109	109
Cross-lingual variation	247	214	214
Explicitly mentioned symptoms	58	67	67
Symptom overgeneralization	71	79	79
